# Linking fatty acid composition to enzyme inhibition supported by molecular docking, alongside multifunctional bioactivities, of avocado, Indian mustard, and passion fruit seed oils

**DOI:** 10.3389/fnut.2026.1874793

**Published:** 2026-07-16

**Authors:** Florinda Fratianni, Giuseppe Amato, Maria Neve Ombra, Francesca Coppola, Beatrice De Giulio, Raffaele Coppola, Gokhan Zengin, Jesus Fernando Ayala Zavala, Laura De Martino, Vincenzo De Feo, Filomena Nazzaro

**Affiliations:** 1 Institute of Food Sciences, CNR-ISA, Avellino, Italy; 2 Department of Pharmacy, University of Salerno, Fisciano, Italy; 3 Department of Agricultural Sciences, University of Naples Federico II, Portici, Italy; 4 Department of Agricultural, Environmental and Food Sciences (DiAAA), University of Molise, Campobasso, Italy; 5 Department of Biology, Faculty of Science, Selcuk University, Konya, Türkiye; 6 Centro de Investigación en Alimentación y Desarrollo, A.C. (CIAD), Carretera Gustavo Enrique Astiazarán Rosas, Hermosillo, Sonora, Mexico

**Keywords:** anti-biofilm, anti-inflammatory, antioxidant, edible oils, fatty acids, neurodegenerative, probiotics

## Abstract

**Introduction:**

The fatty acid composition and multifunctional biological activities of seed oils from *Brassica juncea* (L.) Czern. (Indian mustard), *Passiflora edulis* Sims (passion fruit), and *Persea americana* Mill. (avocado) were investigated. GC–MS analysis revealed distinct lipid profiles among the oils.

**Results and discussion:**

Avocado seed oil was dominated by oleic acid (92.37%), whereas Indian mustard oil contained high levels of erucic acid (48.23%) and α-linolenic acid (18.29%). Passion fruit seed oil was characterized by linoleic acid (54.03%), elaidic acid (20.04%), and stearic acid (15.85%). The unsaturated/saturated fatty acid ratio was highest in avocado oil (18.57), followed by Indian mustard (13.93) and passion fruit oil (3.81). Biological assays revealed differentiated functional properties. Passion fruit oil exhibited the strongest antioxidant activity, with a DPPH IC_50_ of 2.55 μg/mL and showed the highest butyrylcholinesterase inhibition (63.3%). Indian mustard oil demonstrated the greatest anti-arthritic activity (IC_50_ = 6.61 μg) and a high ABTS antioxidant capacity (5.18 μmol TE/g). Avocado oil displayed notable acetylcholinesterase inhibition (58.8%) and selective tyrosinase inhibitory activity (IC_50_ = 13.95 μg). Antibiofilm assays showed strain- and stage-dependent effects, with avocado oil producing a consistent inhibition of mature biofilm structure (77.80% against *Listeria monocytogenes*, and 68.76% against *Pseudomonas aeruginosa*) and inhibitory activity against microbial sessile cells, reaching 47.93% against *P. aeruginosa* and 53.63% against *Escherichia coli*. All three oils positively influenced the growth of selected probiotic strains. Molecular docking, correlation analysis, and principal component analysis highlighted distinct relationships between fatty acid composition and biological activities, suggesting complementary functional profiles and application- specific potential. This study provides the first comparative evaluation of the fatty acid composition, enzyme inhibitory properties, antibiofilm activity, and probiotic-supporting effects of these three seed oils, highlighting their potential as multifunctional ingredients for food, nutraceutical, cosmetic, and health-related applications.

## 1 Introduction

Seed oils obtained from underutilized agro-industrial by-products present significant opportunities for sustainable valorization (1). In contrast to conventional vegetable oils, seed oils derived from fruit-processing residues remain largely unexplored, despite their high concentrations of bioactive compounds, including polyunsaturated fatty acids. These compounds exhibit a broad spectrum of biological activities, including antioxidant, anti-inflammatory, neuroprotective, and antimicrobial effects, making them attractive candidates for therapeutic, nutraceutical, and functional food applications (2). Furthermore, the recovery of oils from seed by-products contributes to sustainable resource management, reduces environmental burdens associated with agro-industrial waste disposal, and promotes the development of environmentally friendly technologies aligned with circular economy principles (3, 4). In recent years, increasing attention has been devoted to the biological and technological properties of seed oils, highlighting their potential as multifunctional ingredients in food, pharmaceutical, nutraceutical, and cosmetic formulations (5, 6). Among their biological properties, antioxidant activity is particularly important because it contributes to the prevention of oxidative stress-related disorders and enhances the stability and shelf life of food products (7, 8). Anti-inflammatory effects, frequently associated with polyunsaturated fatty acids and other bioactive constituents, suggest potential applications in the prevention and management of chronic inflammatory conditions (9, 10). Neuroprotective properties, especially those related to the modulation of cholinesterase enzymes, have attracted considerable interest due to their relevance in neurodegenerative diseases such as Alzheimer’s disease (11, 12). In addition, enzyme inhibitory activities, including tyrosinase inhibition, have gained increasing attention because of their potential applications in dermatology and cosmetic formulations aimed at controlling hyperpigmentation and other skin disorders (13, 14). Despite the growing body of evidence supporting the health-promoting properties of seed oils, comprehensive comparative studies evaluating multiple oils under standardized experimental conditions remain scarce. Consequently, the relative contribution of their fatty acid composition to specific biological activities and the underlying structure–activity relationships are still not fully understood. This study examines three phylogenetically and compositionally distinct seed oils: *Passiflora edulis* Sims (Passifloraceae, passion fruit), *Persea americana* Mill. (Lauraceae, avocado), and *Brassica juncea* (L.) Czern. (Brassicaceae, brown/Indian mustard). The selection of these species was based on both scientific and practical considerations. They belong to distinct botanical families and produce oils characterized by markedly different fatty acid profiles and bioactive constituents, enabling the investigation of possible structure–activity relationships. In addition, all three species are associated with agro-industrial processing streams that generate valuable seed-derived by-products, including passion fruit seeds (15), avocado seeds (16), and Indian mustard meal (17), which represent promising sources of bioactive compounds. Despite increasing interest in the biological properties of these oils, comparative studies performed under standardized experimental conditions remain limited. Therefore, the present work was designed to address this knowledge gap through a comparative evaluation of three chemically distinct seed oils.

*P. edulis* Sims, a tropical vine in the Passifloraceae family, is cultivated worldwide for its aromatic and nutritionally valuable fruit. Among approximately 500 *Passiflora* species, *P. edulis* is particularly notable for its economic importance and medicinal potential (18). The seeds, which are generally discarded as processing by-products, represent a valuable source of oil rich in unsaturated fatty acids, especially linoleic acid, together with tocopherols, phytosterols, and phenolic compounds (6). The composition of passion fruit seed oil has been reported to vary considerably depending on cultivar, geographical origin, environmental conditions, extraction method, and analytical approach (19, 20). Recent studies have highlighted its antioxidant, anti-inflammatory, cholinesterase-inhibitory, antimicrobial, and wound-healing-related properties, supporting its potential application in nutraceutical, pharmaceutical, and dermo-cosmetic formulations (20, 21).

*P. americana* Mill, a member of the Lauraceae family native to Mesoamerica, has attracted significant scientific interest due to its unique lipid composition. While most fruit oils are extracted from seeds, avocado oil is primarily obtained from the mesocarp. However, the seed oil, which contains approximately 2% lipids, remains underutilized and possesses distinct bioactive properties (22). Avocado seed oil is notable for its high monounsaturated fatty acid content, predominantly oleic acid (23, 24), together with tocopherols, carotenoids (particularly lutein), and an unsaponifiable fraction rich in phytosterols, especially β-sitosterol, which have been associated with anti-inflammatory and cholesterol-modulating effects (25). Although oleic acid has consistently been reported as the major fatty acid in avocado-derived oils, its relative abundance may vary considerably depending on cultivar, geographical origin, environmental conditions, seed maturity, extraction procedures, storage conditions, and analytical methodologies. Such compositional variability highlights the importance of characterizing individual samples when evaluating the biological potential and functional properties of avocado seed oil. Avocado seed extracts have antioxidant, anti-hyperglycemic, hepatoprotective, and anti-inflammatory activities, with phenolic compounds contributing significantly to these effects (26). The unsaponifiable fraction has also been studied for its potential in managing osteoarthritis (27).

*B. juncea* (L.) Czern., a member of the Brassicaceae family, is a widely cultivated oilseed crop valued for both its pungent condiment and high-quality oil. Its seed oil is characterized by a unique phytochemical composition and a balanced fatty acid profile, containing approximately 60% monounsaturated fats and 21% polyunsaturated fats, including ω-3 fatty acids (28). The oil also contains tocopherols, carotenoids (lutein and β-carotene), and phytosterols (29). Traditionally, this plant has been used in Asian medicine to treat inflammatory conditions, arthritis, and microbial infections (17). Contemporary research has confirmed its antimicrobial, anti-inflammatory, antioxidant, and potential cardioprotective properties, primarily attributed to allyl isothiocyanate and sinapic acid derivatives (30). Although several studies have investigated the biological properties of plant oils, the relationship between fatty acid composition and antibiofilm activity remains poorly understood, particularly when antioxidant, enzymatic, and antibiofilm properties are evaluated simultaneously.

The aim of this study was to investigate the fatty acid profiles of *B. juncea, P. edulis*, and *P. americana* seed oils, along with a comparative evaluation of their antioxidant, anti-inflammatory (via inhibition of protein degradation), neuroprotective (via inhibition of acetylcholinesterase and butyrylcholinesterase), and tyrosinase inhibitory properties. The inhibitory effect of the oils on immature and mature biofilm produced by different pathogenic strains, as well as their effect on the sessile cells ‘metabolism, was also evaluated. Finally, we evaluated whether they could influence the growth of some probiotic strains. By combining biochemical profiling with *in vitro* functional tests conducted under standardized conditions, this study seeks to deepen understanding of the multifunctional capacities of these seed oils and to provide evidence-based recommendations for their use as natural ingredients in food, nutraceutical, cosmetic, and pharmaceutical formulations.

## 2 Materials and methods

### 2.1 Oils

Three organic commercial seed oils of *Brassica juncea* (L.) Czern (Mystic Moments, Fordingbridge, Hants, United Kingdom), *Passiflora edulis* Sims (Stelcore Retail, Paris, France), and *Persea americana* Mill (AromaLabs, Lyon, France) were used for the experiments. As specified by the manufacturers, the seeds were cold-pressed, avoiding solvents. Samples were stored in sealed amber glass containers at 20 °C, protected from direct light exposure, and analyzed within 2 days after purchase to minimize oxidative degradation and preserve oil quality.

### 2.2 Fatty acid analysis

Fatty acid methyl esters (FAMEs) were prepared by transmethylation following the method of El Riachy et al. (31). Gas chromatographic analysis was conducted using an Agilent 6850 Series II apparatus (Agilent, Roma, Italy) equipped with an HP-5MS capillary column (30 mm × 0.25 mm, 0.25 μm film thickness, Agilent, Roma, Italy). Helium served as the carrier gas at a flow rate of 1 mL/min. FAMEs (1 μL, 10% in CH_2_Cl_2_, v/v) were injected in split mode (50:1). The injector temperature was maintained at 250 °C, while detector temperatures were set at 280 °C for the flame ionization detector (FID) and 180 °C for mass spectrometry (MS, Agilent Mass Selective Detector MSD 5973; Agilent, Roma, Italy). For GC-MS analysis, the ionization voltage, electron multiplier voltage, and ion source temperature were set to 70 eV, 900 V, and 230 °C, respectively. The elution program consisted of an initial temperature of 220 °C for 6 min, followed by a 3 °C/min increase to 270 °C, and a final hold at 270 °C for 4 min. Compounds were identified by calculating their Kovats retention indices relative to the reference standard. All analyses were conducted in triplicate, and results are presented as mean ± standard deviation (SD) of three experiments.

### 2.3 Antioxidant activity

Antioxidant properties were evaluated according to the procedure described by Fratianni et al. (32). Oils were mixed with acetone (Sigma, Milan, Italy) in a 1:1 (v/v) ratio. After incubation for 1 h at room temperature, a 1% methanol-HCl solution (1:2, v/v) was added to each mixture. Samples were then incubated at room temperature for an additional hour and centrifuged at 13,000 rpm for 5 min. The supernatant was collected, and the remaining pellet was subjected to a second extraction using the same protocol. Both supernatants were combined for all subsequent analyses.

#### 2.3.1 DPPH radical scavenging assay

The radical scavenging capacity of the oils was determined using the DPPH (2,2-diphenyl-1-picrylhydrazyl) assay, adapted for microplate analysis (33). Samples were diluted in methanol and combined with 303 μL of DPPH methanolic solution (153 mM). Absorbance was measured at 517 nm (Cary Varian, Palo Alto, CA, United States). The DPPH solution without the sample served as the control. The IC_50_ value, defined as the amount of sample (μg) required to inhibit 50% of DPPH radicals per 1 mL of solution, was calculated. All experiments were conducted in triplicate, and results are reported as mean ± standard deviation (SD).

#### 2.3.2 ABTS radical cation decolorization assay

The antioxidant activity of the oils was evaluated using the ABTS [2,2′-azino-bis (3-ethylbenzothiazoline-6-sulfonic acid)] assay (34). A freshly prepared 2.5 mM Trolox^®^ solution in methanol (Sigma-Aldrich Italia, Milan, Italy) served as the antioxidant reference standard. ABTS and potassium persulfate were dissolved separately in distilled water to final concentrations of 7 mM and 2.45 mM, respectively. The mixture was incubated in the dark at room temperature to generate the ABTS radical cation (ABTS + ). This radical solution was diluted with deionized water to an absorbance of 1.00 at 734 nm. Test samples (final concentrations 0.0001–0.0100 mg/mL) or Trolox^®^ standards (0–20 mM) were added to the diluted ABTS + solution, and absorbance was measured (Cary Varian, Palo Alto, CA, United States) after 6 min incubation. Results are presented as mean ± standard deviation from three independent experiments and expressed as Trolox^®^ equivalents per gram of oil (μmol TE/g).

### 2.4 Nutritional lipid indices

To further evaluate the nutritional quality and oxidative susceptibility of the investigated oils, the hypocholesterolemic/hypercholesterolemic ratio (HH) and the calculated oxidizability value (COX) were determined. A simplified HH ratio as an indicator of the nutritional quality of the lipid fraction based on the proportion of unsaturated to saturated fatty acids was calculated according to the concept proposed by Santos-Silva et al. (35). The COX value was calculated according to Fatemi and Hammond (36) using the following equation:


COX=[(1×%C18:1)+(10.3×%C18:2)

    +(21.6×%C18:3)]/100

The COX index reflects the theoretical susceptibility of oils to oxidative degradation and should therefore be interpreted as an indicator of oxidative stability rather than a nutritional index.

Higher HH values indicate a more favorable nutritional lipid profile, whereas higher COX values reflect greater susceptibility of the oil to oxidative degradation.

### 2.5 *In vitro* anti-inflammatory activity

*In vitro* anti-arthritic activity was assessed using the bovine serum albumin (BSA) denaturation assay (37, 38). Each reaction mixture (total volume 5 mL) contained 0.2 mL of 0.5% (w/v) BSA solution (purity 96%, Sigma, Milan, Italy) prepared in 0.05 M Tris-Phosphate buffered saline (pH 6.5), 2.8 mL of the same buffer, and 2 mL of the sample at various concentrations. A control sample containing BSA dissolved in methanol was included. Mixtures were heated to 72°C, cooled to room temperature, and absorbance was measured at 660 nm (Cary Varian, Palo Alto, CA, United States). The IC_50_ value, representing the concentration required to inhibit 50% of BSA denaturation, was determined. Diclofenac sodium (IC_50_ = 5.5 μg) was used as the positive control. All assays were performed in triplicate, and results are reported as mean ± standard deviation.

### 2.6 Cholinesterase inhibition activities

The inhibitory activities against acetylcholinesterase (AChE) and butyrylcholinesterase (BChE) were evaluated *in vitro* using the spectrophotometric method of Ellman et al. (39). For the AChE inhibition assay, acetylcholine (ACh) served as the substrate. Each reaction mixture contained 550 μL of 0.1 M sodium phosphate buffer (pH 8.0), 50 μL of the test sample, and 5–20 ng of AChE (from *Electrophorus electricus*, 1,000 U/mg). Both test samples and the positive control (galantamine at 10 μM, Sigma-Aldrich, Milan, Italy) were prepared in 10% DMSO. After a 15-min incubation at room temperature, 10 μL of 5,5′-dithiobis (2-nitrobenzoic acid) (DTNB) and 10 μL of ACh were added. The formation of the yellow 5-thio-2-nitrobenzoate anion was monitored by measuring absorbance at 412 nm over 10 min (Cary Varian, Palo Alto, CA, United States). For the BChE inhibition assay, butyryl-thiocholine (BCh) was used as the substrate. The reaction mixture consisted of 550 μL of 0.1 M potassium phosphate buffer (pH 7.0), 50 μL of the test sample, and 10–50 ng of BChE (from equine serum, ≥10 U/mg). After a 15-min incubation at room temperature, 10 μL each of DTNB and BCh were added, and absorbance at 412 nm was measured after 10 min (Cary Varian, Palo Alto, CA, United States). The IC_50_ value, defined as the sample concentration required to inhibit 50% of enzyme activity, was calculated. All results are presented as mean ± standard deviation from three independent replicates.

### 2.7 Tyrosinase inhibition assay

Tyrosinase inhibitory activity was assessed *in vitro* following the method of Khatib et al. (40), with minor modifications. Test samples were dissolved in DMSO (Sigma-Aldrich, Milan, Italy). In 96-well microplates, 70 μL of phosphate buffer (pH 6.8), tyrosinase enzyme (10 U/mL), and the sample were combined and incubated at 37 °C for 5 min. Subsequently, 0.5 mM L-dopamine (DOPA) was added, and after an additional 10-min incubation at 37 °C, absorbance was measured at either 492 or 475 nm (Cary Varian, Palo Alto, CA, United States). Kojic acid served as the positive control, and phosphate buffer was used as the blank. The IC_50_ value, representing the concentration required to inhibit tyrosinase activity by 50%, was determined from the dose–response curve. Each measurement was performed in triplicate, and results are reported as mean ± standard deviation.

### 2.8 Molecular docking analysis of fatty acids identified in the seed oils

To support the interpretation of the enzyme inhibitory assays, an *in silico* docking analysis was carried out using the fatty acids identified by GC–MS in avocado, Indian mustard, and passion fruit seed oils. The analytical profile of the oils showed that avocado oil was mainly composed of oleic acid, Indian mustard oil was dominated by erucic and α-linolenic acids, and passion fruit oil was enriched in linoleic, elaidic, and stearic acids. Based on this composition, the following identified fatty acids were considered for docking analysis: oleic, stearic, gondoic, behenic, lignoceric, erucic, α-linolenic, linoleic, nervonic, and elaidic acids. Eicosenoic acid was reported in the chromatographic dataset but excluded from docking because its positional isomer was unspecified.

Docking simulations were performed against enzyme systems selected to match the *in vitro* assays described in the study: acetylcholinesterase (AChE), butyrylcholinesterase (BChE), and tyrosinase. For AChE, two receptors were used: human AChE in the galantamine-binding gorge (PDB: 4EY6) and *Electrophorus electricus* AChE in the homologous catalytic gorge (PDB: 1EEA). For BChE, the catalytic pocket of human BChE (PDB: 1P0I) was used as a structural surrogate for the equine serum BChE employed in the *in vitro* assay. For tyrosinase, two regions were evaluated in *Bacillus megaterium* tyrosinase (PDB: 3NQ1): the dicopper catalytic site and a kojic-acid-like adjacent pocket. These target choices were aligned with the wet-lab assays, which used acetylcholine as substrate and galantamine as positive control for AChE, butyrylthiocholine for BChE, and L-DOPA with kojic acid as positive control for tyrosinase.

For each receptor–ligand pair, three independent AutoDock Vina simulations were performed using different random seeds. Each run used exhaustiveness = 16, num_modes = 9, and energy_range = 4, and the best score from each run was retained. Results were expressed as mean ± standard deviation. Thus, the reported values integrate independent stochastic runs and internal pose exploration, although the number of independent simulations remains limited compared with more extensive structure-based optimization protocols. Docking was therefore interpreted as a complementary, hypothesis-supporting analysis rather than as definitive proof of binding or inhibition. In addition to the docking score, recurrent protein residues located in the binding region were recorded to support site-level interpretation. Binding poses were interpreted according to their location within the catalytic gorge, the gorge/peripheral transition region, the BChE catalytic pocket, the tyrosinase dicopper site, or the adjacent kojic-acid-like pocket. The final ranking of candidate fatty acids was based on a combined criterion including docking score consistency across triplicate runs, recurrence of interactions with catalytically relevant residues, ligand localization within catalytic or adjacent binding regions, and the actual abundance of each fatty acid in the corresponding oil.

### 2.9 Evaluation of antimicrobial properties

The bacterial strains used in this study were *Acinetobacter baumannii* ATCC 19606, *Escherichia coli* DSM 8579, a clinical isolate of *Klebsiella pneumoniae*, *Listeria monocytogenes* ATCC 7644, *Pseudomonas aeruginosa* DSM 50071, and *Staphylococcus aureus* subsp. *aureus* Rosenbach ATCC 25923. All strains were cultured in Luria–Bertani broth for 18 h under aerobic conditions at 37 or 35 °C, as appropriate for each strain, with shaking at 80 rpm (Corning LSE, Pisa, Italy) prior to experimentation. Resazurin, crystal violet, MTT, and dimethyl sulfoxide (DMSO) were sourced from Sigma-Aldrich (Milan, Italy).

#### 2.9.1 Determination of minimum inhibitory concentration (MIC)

Minimum inhibitory concentration (MIC) was determined using a resazurin-based microtiter plate assay (41) in flat-bottom 96-well plates. Following incubation at 37 °C for 24 h (or 35 °C for *A. baumannii*), bacterial growth was assessed by monitoring the color change of resazurin. Sterile DMSO served as the negative control, and tetracycline (1 mg/mL in DMSO; Sigma-Aldrich) as the positive control. Each assay was conducted in triplicate, and results are reported as mean ± standard deviation.

#### 2.9.2 Impact on biofilm formation

The impact of seed oils on bacterial biofilm formation was assessed using the crystal violet (CV) staining method (Sigma-Aldrich, Italy) in flat-bottom 96-well microplates (Falcon, VWR International, Milan, Italy) (42). In brief, 10 μL of an overnight bacterial culture, standardized to 0.5 McFarland, in fresh LB broth, was added to each well, along with 10 or 20 μL/mL of the oils and sterile LB medium, for a final volume of 250 μL. Plates were sealed with parafilm to minimize evaporation and incubated for 48 h at 37 or 35 °C, as appropriate. After incubation, planktonic cells were removed, and wells were washed twice with sterile PBS. Adherent cells were fixed with 200 μL methanol for 15 min, after which methanol was discarded, and plates were air-dried. Wells were stained with 200 μL of 2% (w/v) CV solution for 20 min. Excess dye was removed, and wells were rinsed with sterile PBS and allowed to dry. The bound CV was solubilized with 200 μL of 20% (w/v) glacial acetic acid, and absorbance was measured at 540 nm (Cary Varian). Inhibition of biofilm formation was quantified as a percentage relative to the untreated control (set as 0% inhibition). Each experiment was performed in triplicate, and results are reported as mean ± SD.

#### 2.9.3 Effects on established biofilms

To assess activity against mature biofilms, 10 μL of an overnight bacterial culture at 0.5 McFarland in LB broth was added to 96-well plates, bringing the final volume to 250 μL per well. Plates were sealed and incubated for 24 h at 37 or 35 °C, as appropriate for each strain. After removal of planktonic bacteria, oils were added at final concentrations of 10 and 20 μg/mL, and fresh LB broth was added to maintain a total volume of 250 μL. Plates were incubated for an additional 24 h. Biofilm inhibition was determined relative to untreated mature biofilms, as described above.

#### 2.9.4 Effect of the seed oils on the metabolic activity of sessile cells

The metabolic activity of sessile bacterial cells in the presence of oils was assessed using the MTT [3-(4,5-dimethylthiazol-2-yl)-2,5-diphenyltetrazolium bromide] assay (Sigma-Aldrich, Italy) (43). Oils were evaluated at concentrations of 10 and 20 μL/mL, added either at the initiation of bacterial growth or after 24 h to examine effects on both developing and mature biofilms. After a total incubation period of 48 h, planktonic cells were removed, and 150 μL of PBS and 30 μL of 0.3% (w/v) MTT solution were added to each well. Plates were incubated for 2 h at 37 or 35 °C, as appropriate. The MTT solution was then discarded, and wells were rinsed twice with 200 μL sterile PBS. Formazan crystals were dissolved with 200 μL DMSO, and absorbance was measured at 570 nm (Cary Varian, Palo Alto, CA, United States).

### 2.10 Impact of the oils on the probiotic growth

*Lactobacillus gasseri* LG050, and *Lacticaseibacillus paracasei subs paracasei* I 1688 were bought as commercial formulations available from a local pharmacy; *Lactiplantibacillus plantarum* subsp. *plantarum* DSM 20,174, and *Lactobacillus delbrueckii* subsp. *bulgaricus* DSM 20,081 were purchased by Deutsche Sammlung von Mikroorganismen und Zellkulturen GmbH, Braunschweig, Germany. Cells were anaerobically grown at 37 °C (except *L. plantarum*, grown at 30°C) for 16–18 h in MRS (Liofilchem, Roseto degli Abruzzi, Italy). Into the medium, 20 mL/mL of the seed oils was added. The growth was read at λ = 600 nm (Cary 50Bio, Varian, Palo Alto, CA, United States). The effect of the three seed oils on lactic bacterial growth was calculated as a percentage relative to the control, with the strains grown in the presence of glucose.

### 2.11 Statistical analysis

Results are reported as mean ± standard deviation from three independent experiments (PC software “Excel Statistics” version 365). One-way analysis of variance (ANOVA) was performed, followed by Tukey’s *post-hoc* test.

Principal component analysis (PCA) was performed to explore the relationships between fatty acid composition and the biological activities of the investigated seed oils. The dataset included variables related to the fatty acid profile (e.g., oleic acid, linoleic acid, linolenic acid, erucic acid, total saturated fatty acids, total unsaturated fatty acids, and the unsaturated/saturated fatty acid ratio) as well as biological activity parameters, such as antioxidant activity (DPPH IC_50_ and ABTS values), anti-arthritic activity, acetylcholinesterase (AChE) inhibition, butyrylcholinesterase (BChE) inhibition, tyrosinase inhibition, and anti-biofilm activity. Prior to PCA, all variables were standardized using z-score normalization to eliminate scale differences among the measured parameters. PCA was then performed on the standardized dataset using a covariance-based approach, and the first two principal components (PC1 and PC2) were retained for visualization and interpretation. Pearson correlation coefficients (r) were calculated to investigate the relationship between fatty acid composition and bacterial growth. These statistical analyses were carried out using Python (version 3.x) with the scikit-learn library, and graphical outputs were generated using the Matplotlib library. Pearson correlation coefficients were calculated among compositional parameters (percentages of major fatty acids, saturated fatty acids (SFAs), unsaturated fatty acids (UFAs), and the UFAs/SFAs ratio), measured antibiofilm activities from crystal violet (CV) and MTT assays, and probiotic growth parameters. For the antibiofilm parameters, the analysis was conducted separately for each pathogenic strain and for each experimental condition (immature and mature biofilm) at the highest concentration (20 μL/mL) used in the antibiofilm activity tests. For the probiotics growth results, the analysis was performed separately for each bacterial strain. For each combination of lipid parameter, antibiofilm activity, and prebiotic activity, the values corresponding to the three vegetable oils were used. The *r*-values range from −1 to + 1, indicating perfect negative and positive correlations, respectively. The same correlation analysis was applied to investigate the relationships between fatty acid composition and other biological activities, including antioxidant, anti-arthritic, and neuroprotective effects. For each activity, Pearson correlation coefficients were calculated using values obtained for the three oils and the corresponding lipid parameters, as described above.

## 3 Results and discussion

### 3.1 Fatty acid composition

The lipid profiles of the three seed oils (Table 1) revealed substantial differences. Passion fruit oil contained a high proportion of linoleic acid (54.03% ± 3.64), followed by elaidic acid (20.04% ± 2.21) and stearic acid (15.85% ± 2.45), resulting in an unsaturated to saturated fatty acid ratio of 3.81. Indian mustard seed oil was dominated by erucic acid (48.23% ± 3.61) and linolenic acid (18.29% ± 1.15), with moderate levels of linoleic acid (12.57% ± 0.95) and gondoic acid (9.90% ± 1.62). Avocado seed oil was characterized by an extremely high proportion of monounsaturated fatty acids (ΣMUFAs = 94.17%). It exhibited a predominance of oleic acid (92.37% ± 5.57), with only minor amounts of stearic (4.09% ± 0.94) and gondoic (1.25% ± 0.31) acids. The oleic acid content detected in the present study is higher than commonly values reported for avocado seed oil (23, 24). However, it has been described as a major fatty acid in avocado-derived oils Although our analysis revealed a considerably higher oleic acid content than that typically reported for avocado seed oil, it is important to note that the literature consistently identifies oleic acid as the predominant fatty acid in avocado-derived oils. For instance, Otaigbe et al. (23) reported oleic acid levels of approximately 62.69%, whereas Gidigbi et al. (24) described an oleic acid content of 67.80%. Substantial variability in oleic acid content has been documented in relation to cultivar, geographical origin, environmental conditions, extraction procedures, and analytical methodologies (25). Therefore, the fatty acid profile observed in the present study is likely representative of the specific commercial sample analyzed rather than universally representative of avocado seed oils.

**TABLE 1:** Fatty acid composition (%) of the seed oils of avocado, Indian mustard, and passion fruit.

Fatty acid	Avocado seed oil (%)	Indian mustard seed oil (%)	Passion fruit seed oil (%)
Linolenic acid (C18:3 w-3)	–	18.29 ± 1.15	–
Linoleic acid (C18:2 w-6)	–	12.57 ± 0.95	54.03 ± 3.64
Oleic acid (C18:1 w-9)	92.37 ± 5.57	–	–
Elaidic acid (trans-C18:1)	–	–	20.04 ± 2.21
Stearic acid (C18:0)	4.09 ± 0.94	1.52 ± 0.78	15.85 ± 2.45
Gondoic acid (C20:1 w-9)	1.25 ± 0.31	9.90 ± 1.62	1.73 ± 0.25
Eicosenoic acid (C20:1)	0.55 ± 0.07	1.60 ± 0.42	3.35 ± 0.19
Erucic acid (C22:1 w-9)	–	48.23 ± 3.61	–
Behenic acid (C22:0)	0.16 ± 0.05	2.43 ± 0.69	2.15 ± 0.85
Nervonic acid (C24:1)	–	3.37 ± 0.78	1.07 ± 0.11
Lignoceric acid (C24:0)	0.24 ± 0.09	1.08 ± 0.45	0.95 ± 0.13
Total	98.66	98.99	99.17
w-6/w-3	nc	1.45	nc
Σ MUFAs	94.17	63.10	26.19
Σ PUFAs	–	30.86	54.03
SFAs	5.04	6.63	20.15
UFAs	93.62	92.36	76.87
UFAs/SFAs ratio	18.57	13.93	3.81

Data are the average of three different measures ± standard deviation (SD). Total MUFAs, Total PUFAs, Total SFAs, total UFAs, the ω-6/ω-3 ratio, and the UFAs/SFAs ratio, are derived values calculated from the corresponding mean fatty acid composition and do not represent independent experimental measurements. Therefore, standard deviations were not reported for these calculated indices. –, not detected; nc, not calculable.

Coppola et al. (42) identified linoleic acid as the principal component of passion fruit seed oil; Similarly, Chakroborty et al. (44) reported the presence of erucic acid in Indian mustard seed oil, which was also observed in the present sample. Indian mustard oil exhibited a mixed profile with substantial amounts of both ΣMUFAs (63.10%) and ΣPUFAs (30.86%). Both Indian mustard and avocado oils showed higher unsaturated-to-saturated fatty acid ratios (13.93 and 18.57, respectively) than passion fruit oil, indicating a greater overall degree of unsaturation. Additionally, Indian mustard oil exhibited an ω-6/ω-3 ratio of approximately 1:1, as reported in the literature (45). From a nutritional perspective, the fatty acid profiles of these oils are of particular interest due to the well-established health benefits of diets rich in unsaturated fatty acids and a balanced ω-6/ω-3 ratio. High intake of monounsaturated fatty acids (MUFAs), such as oleic acid found in avocado seed oil, has been linked to improved lipid profiles, reduced low-density lipoprotein (LDL) cholesterol, and a decreased risk of cardiovascular disease (46, 47). Similarly, polyunsaturated fatty acids (PUFAs), including linoleic acid (an ω-6 PUFA) and α-linolenic acid (an ω-3 PUFA), are essential for human health. In fact, they play critical roles in modulating inflammatory responses and membrane fluidity, and in preventing chronic diseases (48, 49). For cardiovascular well-being, oils that contain a high proportion of unsaturated fatty acids—such as avocado oil and Indian mustard seed oil—are considered beneficial (50). The ω-6/ω-3 fatty acid ratio is another important nutritional parameter. An excessive dietary ratio, as commonly found in Western diets, has been associated with an increased risk of inflammatory, metabolic, and cardiovascular diseases (48, 51). Conversely, an approximate balance of ω-6 and ω-3 fatty acids, as observed in Indian mustard seed oil, is considered optimal for health promotion and disease prevention. Evidence suggests that the optimal ω-6/ω-3 ratio may vary by disease context and genetic factors, but a lower ratio is generally favorable for reducing the risk of prevalent chronic diseases, particularly those increasingly associated with Western dietary patterns worldwide (52). Furthermore, the overall low content of saturated fatty acids in these oils—especially in avocado and Indian mustard seed oils—may confer additional benefits, as high consumption of saturated fats has been linked to the progression of atherosclerosis and other cardiometabolic disorders (53). Notably, the absence or very low levels of medium-chain saturated fatty acids (C12:0, C14:0, and C16:0) in these oils may further enhance their nutritional value, given the established association of these fatty acids with elevated plasma cholesterol levels (54). Avocado oil showed the highest HH value (18.58), followed by mustard oil (13.93), while passion fruit oil displayed a lower value (3.82), consistent with its higher saturated fatty acid content. Avocado oil showed the lowest COX value (0.94), reflecting its predominance of monounsaturated fatty acids, mainly oleic acid, which are known to confer higher oxidative stability. In contrast, Indian mustard and passion fruit oils exhibited considerably higher COX values (5.88 and 5.83, respectively), consistent with their higher polyunsaturated fatty acid content. Oils rich in PUFAs are generally more susceptible to oxidative processes, which may influence both their shelf-life stability and their technological applications in food systems.

The fatty acid composition of the three seed oils can be summarized:

Avocado 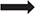 rich in MUFAs high 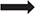 oxidative stability

Indian mustard 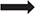 balanced PUFAs 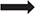 better ω-6/ω-3

Passion fruit 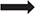 PUFAs-rich oil 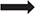 higher sensitivity to oxidation

### 3.2 Antioxidant activity

The biological activities of the three oils are shown in Table 2. The three seed oils demonstrated varying degrees of antioxidant activity, as evidenced by DPPH and ABTS assays. Passion fruit seed oil showed the strongest antioxidant activity, with a DPPH IC_50_ of 2.55 ± 0.09 μg/mL, and 5.25 ± 0.3 μmol TE/g in the ABTS assay.

**TABLE 2:** Biological properties of avocado, Indian mustard, and passion fruit seed oils.

Seed oils	DPPH IC_50_ (μg)	ABTS [μM(TE) /gr]	Anti-arthritic activity IC_50_ (μg)	AChE inhibitory activity (%)	BChE inhibitory activity (%)	Tyrosinase inhibitory activity (DOPA) IC_50_ (μg)
Avocado	7.23^a^ ± 1.03	6.40^a^ ± 0.41	11.01^a^± 1.63	58.80 ^c^ ± 2.28	30.97 ^b^ ± 1.32	13.95^a^ ± 1.04
Indian mustard	4.35^a^ ± 0.67	11.95^a^ ± 0.61	6.61^a^ ± 0.51	23.64 ^a^ ± 1.46	0.00	11.64^a^ ± 0.78
Passion fruit	2.55^a^ ± 0.09	5.25^a^ ± 0.3	12.61^a^ ± 0.88	61.10 ^c^ ± 3.59	63.30 ^c^ ± 2.81	0.00 ± 0.00

Results are the average of three independent experiments ± SD. Different letters over the lines indicate statistically significant differences (*p* < 0.001) according to one-way ANOVA followed by Tukey’s *post-hoc* test.

Oxidative stability is a critical parameter for seed oils from both technological and nutritional perspectives, as it determines shelf life, sensory qualities, and suitability for culinary and industrial applications. Although unsaturated fatty acids, some PUFAs, such as linoleic and linolenic acids, offer significant health benefits, they are more prone to peroxidation than saturated or monounsaturated fatty acids due to their multiple double bonds (55). This increased susceptibility to oxidative degradation can result in the formation of off-flavors and harmful secondary oxidation products during storage or thermal processing (56). The presence of natural antioxidants, including tocopherols, phenolic compounds, carotenoids, and glucosinolate derivatives, can enhance the oxidative stability of seed oils by scavenging free radicals and interrupting lipid peroxidation chains (56, 57). The high radical-scavenging capacity observed in passion fruit and Indian mustard oils likely reflects not only their fatty acid profiles but also the contributions of these minor bioactive constituents. For instance, γ- and δ-tocopherols, which predominate in passion fruit seed oil, have demonstrated greater efficacy in inhibiting lipid oxidation compared to α-tocopherol, particularly in vegetable oils (58). Similarly, the antioxidant performance of Indian mustard oil is likely enhanced by isothiocyanates and phenolic acids, such as sinapic acid, both of which are recognized for their strong electron-donating properties and protective effects against oxidative stress (59). The divergent results between the DPPH and ABTS assays observed in our study highlight the complex interplay between the oils’ chemical composition and their antioxidant mechanisms. DPPH primarily measures hydrogen atom transfer capacity, whereas the ABTS assay captures both hydrogen- and electron-transfer activity, providing a broader assessment of antioxidant potential (60). The relatively high ABTS value for Indian mustard oil suggests the presence of compounds particularly effective at electron transfer, consistent with the known activity of isothiocyanates and phenolic acids (61). Although unsaturated fatty acids promote health, the rapid oxidation of PUFAs underscores the importance of a balanced oil composition. Oils with a predominance of monounsaturated fatty acids, such as avocado oil, generally exhibit greater oxidative stability, whereas those rich in PUFAs, such as passion fruit oil, may require careful storage and the addition of sufficient antioxidants to maintain quality (62). The overall antioxidant capacity of these oils, as demonstrated by both DPPH and ABTS assays, supports their potential as functional ingredients that mitigate oxidative stress and protect against chronic degenerative diseases (63, 64).

### 3.3 *In vitro* anti-inflammatory activity

Denatured proteins can become antigenic, triggering immune responses and biochemical changes in connective tissues that contribute to the onset and progression of rheumatoid arthritis (65). Therefore, agents capable of preventing protein denaturation hold significant promise for novel anti-arthritic therapeutics. The BSA denaturation assay provides a reliable, cost-effective method for screening potential anti-inflammatory and anti-arthritic agents by mimicking *in vivo* protein denaturation (66): in fact, heat-induced protein denaturation mirrors early inflammatory responses (67). This anti-arthritic activity could be attributable to the fatty acids present in these oils. PUFAs, particularly linoleic and α-linolenic acids, significantly contribute to anti-inflammatory and anti-arthritic efficacy (52). Similarly, monounsaturated fatty acids, particularly oleic acid, have been shown to preserve protein structure and reduce prostaglandin concentrations, indicating dual benefits in protein stability and inflammatory modulation (32, 68). In our study, the three seed oils exhibited distinct activities in the *in vitro* BSA denaturation assay (Table 2), with Indian mustard oil showing the most potent inhibitory effect (IC_50_ = 6.61μg ± 0.51), followed by avocado (IC_50_ = 11.01 μg ± 1.63) and passion fruit oils (IC_50_ = 12.61 μg ± 0.88). The lower IC_50_ value of Indian mustard oil indicates superior protein-protective capacity at lower concentrations, suggesting highly effective anti-inflammatory properties. The ability of these fatty acids to inhibit heat-induced protein denaturation is well documented, suggesting that the benefits observed in this study may, at least in part, be attributable to the fatty acid profiles of each oil (69). Furthermore, other research on seed oils demonstrated similar potential by preventing protein denaturation associated with rheumatoid arthritis (68, 70).

### 3.4 Neuroprotective activity

#### 3.4.1 AChE and BChE inhibition

The three seed oils exhibited distinct neuroprotective profiles based on their cholinesterase-inhibitory activities (Table 2). Passion fruit seed oil achieved the most comprehensive dual inhibition, with 61.1% ± 3.58 inhibition of acetylcholinesterase (AChE) and 63.3% ± 2.81 inhibition of butyrylcholinesterase (BChE). Avocado oil exhibited substantial AChE inhibition (58.8% ± 2.28) but lower BChE inhibition (30.97% ± 1.32). Indian mustard oil showed moderate AChE inhibition (23.64% ± 1.45) and no detectable BChE inhibitory activity.

The dual cholinesterase inhibition observed with passion fruit seed oil is particularly significant, as butyrylcholinesterase (BChE) increasingly compensates for acetylcholinesterase (AChE) in sustaining cholinergic neurotransmission during advanced stages of Alzheimer’s disease (71). Most plant-derived oils and lipid fractions typically exhibit selective inhibition of a single cholinesterase (72), underlining the therapeutic relevance of the balanced inhibitory profile of passion fruit seed oil, in contrast to the selective inhibition observed with avocado and Indian mustard oils. This activity suggests that passion fruit seed oil may exert a more robust neuroprotective effect than enzyme-selective inhibitors, potentially mitigating cognitive decline associated with neurodegenerative diseases such as Alzheimer’s disease (72).

The pronounced AChE inhibition and limited BChE activity of avocado oil suggest the presence of bioactive compounds with structural specificity for the AChE catalytic site. This selective inhibition may provide therapeutic benefits during early-stage cognitive decline, when AChE is the predominant cholinesterase, but would likely be less effective in advanced stages of neurodegeneration (73). Indian mustard oil’s modest AChE inhibition and lack of BChE activity indicate a more limited neuroprotective profile than the other oils. Nevertheless, its AChE inhibition may complement its notable anti-arthritic properties, given the recognized interplay between neuroinflammation and peripheral inflammation in neurodegenerative diseases (74).

The observed anticholinesterase activity may be partially attributable to the fatty acid composition identified in this study. PUFAs, especially omega-3 fatty acids, have been linked to increased brain acetylcholine levels and enhanced cognitive function (75). The substantial linoleic acid content in passion fruit seed oil may contribute to its dual inhibitory activity, as certain unsaturated fatty acids can interact with the catalytic or peripheral binding sites of cholinesterases (76). The dual inhibitory activity of passion fruit oil, with inhibition values exceeding 60% for both enzymes, meets established criteria for potent cholinesterase inhibition and suggests that its components may interact with both the catalytic and peripheral anionic sites of these enzymes (77).

Future research employing component-by-component analyses could clarify the precise binding mechanisms and the synergistic effects of fatty acids and other compounds on anticholinesterase activity (78), providing additional information on the therapeutic potential of these oils in neurodegenerative conditions (79).

#### 3.4.2 Tyrosinase inhibitory activity

Tyrosinase catalyzes the rate-limiting step in melanin biosynthesis by converting L-tyrosine to L-DOPA, which is subsequently converted to dopaquinone (80). Inhibition of tyrosinase is relevant for cosmetic applications, such as the treatment of hyperpigmentation disorders and skin whitening, as well as for therapeutic interventions in conditions like melanoma and neurodegenerative diseases involving dopamine metabolism (81).

The three seed oils examined exhibited distinct tyrosinase inhibitory activities, likely attributable to differences in their fatty acid profiles. Both avocado and Indian mustard seed oils demonstrated measurable inhibition of tyrosinase activity, whereas passion fruit seed oil did not display detectable activity. Specifically, avocado oil showed an IC_50_ of 13.95 μg, and Indian mustard oil exhibited a slightly greater potency with an IC_50_ of 11.64 μg (Table 2), when DOPA was used as the substrate. The lack of tyrosinase inhibition by passion fruit seed oil suggests that its fatty acid composition does not confer structural features necessary for enzyme interaction.

These findings highlight the role of fatty acid composition in modulating tyrosinase-inhibitory properties among seed oils. Previous studies have demonstrated that the chain length and degree of unsaturation of fatty acids can influence their interactions with tyrosinase and other biological targets, potentially through hydrophobic interactions or by modulating enzyme conformation (82). Moreover, certain unsaturated fatty acids have been shown to inhibit melanin synthesis and tyrosinase activity (83, 84). The observed differences support the concept that specific structural features within fatty acid chains—such as degree of unsaturation, chain length, and the presence or absence of functional groups—may influence the ability to interact with the enzyme’s active site (84). Further studies are required to clarify the structure–activity relationships and to pinpoint which fatty acid species are primarily responsible for the observed effects.

### 3.5 Docking-based prioritization of fatty acids associated with cholinesterase and tyrosinase inhibition

**FIGURE 1:**
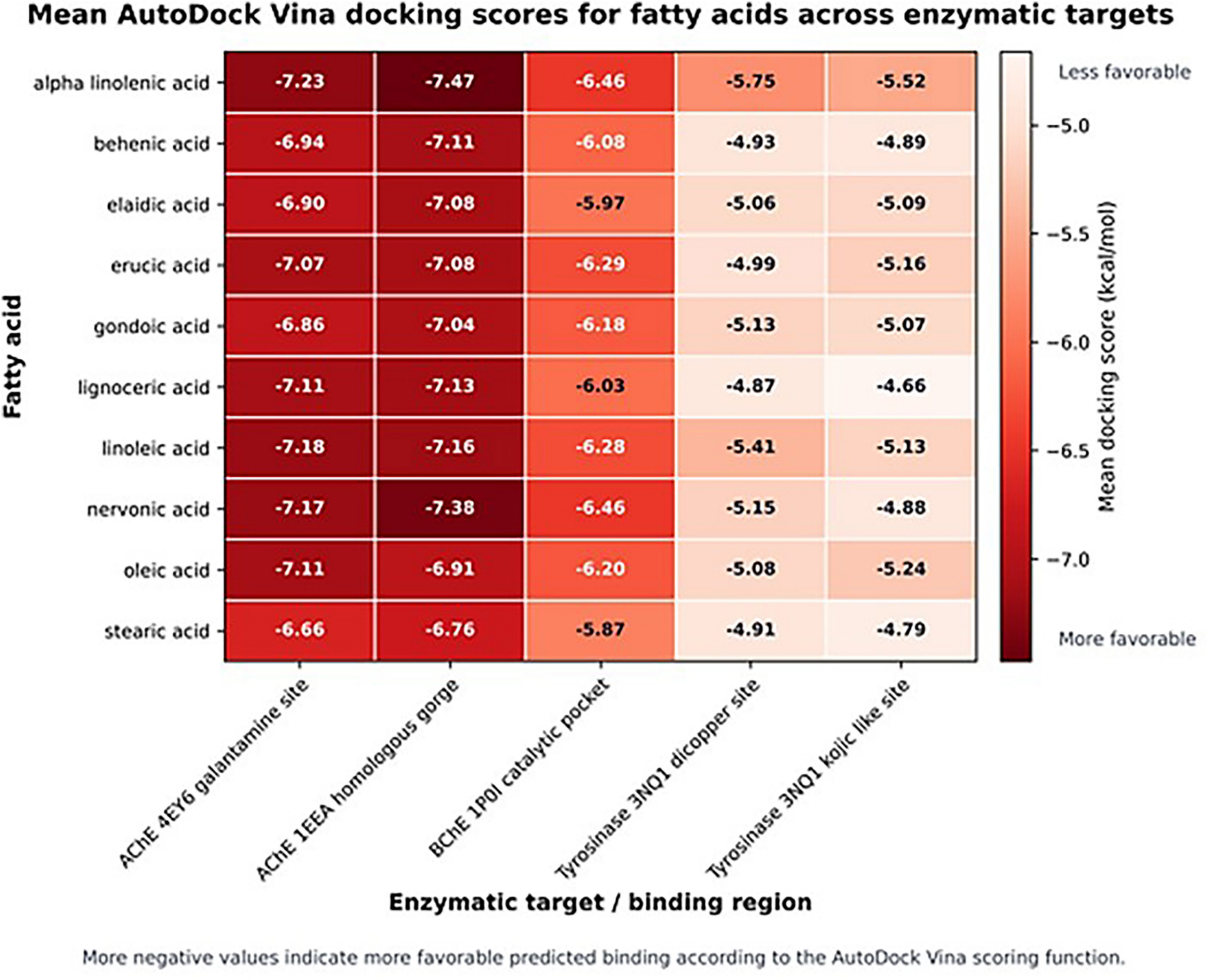
Heatmap summary of the mean AutoDock Vina docking scores across the selected fatty acids and enzymatic targets, allowing direct comparison of relative binding favorability without relying on undefined qualitative categories.

To support the experimental findings, the major and minor fatty acids identified by GC–MS were docked against AChE, BChE, and tyrosinase targets selected to reflect the *in vitro* assays. Results are shown in Table 3 and summarized as a docking-score heatmap in Figure 1. Only fatty acids analytically identified in the three oils by GC–MS were prioritized. Eicosenoic acid, although reported in the chromatographic profile, was not docked because the positional isomer was not specified in the analytical dataset and was therefore excluded to avoid arbitrary structural assignment. Avocado oil was dominated by oleic acid (92.37%), Indian mustard oil by erucic acid (48.23%) and α-linolenic acid (18.29%), and passion fruit oil by linoleic acid (54.03%), elaidic acid (20.04%), and stearic acid (15.85%). The overall pattern seemed consistent across receptors: long-chain unsaturated fatty acids showed the most favorable binding profiles in both cholinesterases, whereas the affinities observed for tyrosinase were more moderate. Among all fatty acids, α-linolenic acid emerged as the most robust candidate, ranking first in 4EY6 AChE (−7.23 ± 0.11 kcal/mol), 1EEA AChE (–7.47 ± 0.08 kcal/mol), and in both tyrosinase regions, namely the dicopper site (−5.75 ± 0.13 kcal/mol) and the kojic-acid-like pocket (−5.51 ± 0.23 kcal/mol). In BChE, nervonic acid (−6.46 ± 0.06 kcal/mol) and α-linolenic acid (−6.46 ± 0.21 kcal/mol) showed nearly identical top scores. Within AChE, the best-ranked fatty acids repeatedly interacted with residues characteristic of the catalytic gorge. In 4EY6, α-linolenic, linoleic, nervonic, lignoceric, and oleic acids contacted residues such as TRP86, TYR124, SER125, PHE297, TYR337, PHE338, and, for some ligands, TRP286 and HIS447, indicating occupation of the catalytic gorge or the gorge/PAS transition. In 1EEA, the same trend was observed, with α-linolenic, nervonic, linoleic, lignoceric, and behenic acids interacting with TRP84, TYR121, SER200, PHE330, PHE331, TYR334, and HIS440. These recurrent contacts support a mechanistically coherent binding mode within the functional AChE gorge.

**TABLE 3:** Molecular docking-based prioritization of fatty acids identified in avocado, Indian mustard, and passion fruit seed oils against cholinesterase and tyrosinase targets.

Target (PDB)	Binding region	Top-ranked fatty acids from the oils	Mean docking score (kcal/mol)	Main recurrent interacting residues	Oil(s) in which the fatty acid was identified
AChE (4EY6)	Galantamine-binding gorge	α-Linolenic acid	−7.23 ± 0.11	TRP86, TYR124, SER125, PHE297, TYR337, PHE338	Indian mustard
AChE (4EY6)	Galantamine-binding gorge	Linoleic acid	−7.18 ± 0.19	TRP86, TYR124, TRP286, PHE297, TYR337, PHE338, TYR341, HIS447	Indian mustard, passion fruit
AChE (4EY6)	Galantamine-binding gorge	Nervonic acid	−7.17 ± 0.10	ASP74, TRP86, TYR124, SER125, GLU202, TRP286	Indian mustard, passion fruit
AChE (1EEA)	Homologous catalytic gorge	α-Linolenic acid	−7.47 ± 0.08	TRP84, TYR121, SER200, PHE330, PHE331, TYR334, HIS440	Indian mustard
AChE (1EEA)	Homologous catalytic gorge	Nervonic acid	−7.37 ± 0.25	TRP84, TYR121, PHE290, PHE330, PHE331, TYR334, HIS440	Indian mustard, passion fruit
AChE (1EEA)	Homologous catalytic gorge	Linoleic acid	−7.15 ± 0.14	TRP84, TYR121, SER122, SER200, PHE330, PHE331, TYR334, HIS440	Indian mustard, passion fruit
BChE (1P0I)	Catalytic pocket	Nervonic acid	−6.46 ± 0.06	TRP82, GLU197, SER198, TRP231, PHE329, TYR332, HIS438, ALA328	Indian mustard, passion fruit
BChE (1P0I)	Catalytic pocket	α-Linolenic acid	−6.46 ± 0.21	TRP82, GLU197, SER198, PHE329, TYR332, HIS438, ALA328	Indian mustard
BChE (1P0I)	Catalytic pocket	Erucic acid	−6.28 ± 0.19	TRP82, SER198, PHE329, TYR332, PHE398, HIS438	Indian mustard
BChE (1P0I)	Catalytic pocket	Linoleic acid	−6.28 ± 0.17	TRP82, GLU197, SER198, TYR128, PHE329, HIS438	Indian mustard, passion fruit
Tyrosinase (3NQ1)	Dicopper catalytic site	α-Linolenic acid	−5.75 ± 0.13	GLY46, HIS60, ASN205, HIS208, GLY216, VAL218, PRO219	Indian mustard
Tyrosinase (3NQ1)	Dicopper catalytic site	Linoleic acid	−5.41 ± 0.07	ASN205, HIS208, GLY216, VAL217, VAL218, PRO219, ALA221	Indian mustard, passion fruit
Tyrosinase (3NQ1)	Dicopper catalytic site	Nervonic acid	−5.15 ± 0.11	GLY46, MET61, PHE197, ASN205, HIS208, ARG209, GLY216	Indian mustard, passion fruit
Tyrosinase (3NQ1)	Kojic-acid-like adjacent pocket	α-Linolenic acid	−5.52 ± 0.23	HIS60, MET61, PHE197, PRO201, HIS204, ASN205, HIS208, ARG209	Indian mustard
Tyrosinase (3NQ1)	Kojic-acid-like adjacent pocket	Oleic acid	−5.24 ± 0.34	HIS60, MET61, PHE197, PRO201, HIS204, ASN205, HIS208, ARG209	Avocado
Tyrosinase (3NQ1)	Kojic-acid-like adjacent pocket	Erucic acid	−5.16 ± 0.13	HIS60, MET61, PHE197, PRO201, HIS204, ASN205, HIS208, ARG209	Indian mustard

Docking scores are reported as mean ± standard deviation from three independent Vina runs per receptor–ligand pair.

For BChE, the highest-ranked fatty acids are also localized in the catalytic pocket. Nervonic, α-linolenic, erucic, linoleic, and oleic acids interacted repeatedly with TRP82, GLU197, SER198, PHE329, TYR332, and HIS438, consistent with occupation of the BChE catalytic gorge. In contrast, tyrosinase yielded lower overall scores. Nevertheless, α-linolenic acid and linoleic acid were the most consistent ligands near the dicopper catalytic environment, where they contacted residues such as HIS60, ASN205, HIS208, GLY216, and VAL218. The kojic-acid-like site behaved as a more peripheral or adjacent pocket, and the larger standard deviations observed there indicate greater pose variability than at the dicopper site. When docking was combined with fatty-acid abundance, Indian mustard oil appeared to have the most promising inhibitory lipid ensemble, as it contains α-linolenic acid, erucic acid, linoleic acid, gondoic acid, and nervonic acid. Passion fruit oil also appeared relevant, mainly because of its high linoleic acid content and the presence of nervonic and elaidic acids. By contrast, avocado oil was compositionally dominated by oleic acid, which showed reproducible but generally less dominant behavior across the enzymatic targets than α-linolenic or linoleic acid. The docking results help refine the interpretation of the experimental enzyme inhibition data by indicating which identified fatty acids are most likely to contribute to the observed bioactivities. A clear structure-related trend emerged: long-chain unsaturated fatty acids performed better than saturated counterparts across most targets, especially in AChE and BChE. This behavior is consistent with the elongated, hydrophobic architecture of cholinesterase gorges, in which flexible aliphatic chains can establish favorable contacts with aromatic and hydrophobic residues while positioning the carboxyl group toward polar regions near the catalytic environment. In both AChE models, α-linolenic acid, linoleic acid, and nervonic acid repeatedly occupied the real gorge region and interacted with residues known to define catalytic or gorge-binding space, supporting the idea that these lipids may contribute to the anticholinesterase activity of the oils.

Figure 1 provides a heatmap summary of the mean AutoDock Vina docking scores across the selected fatty acids and enzymatic targets, allowing direct comparison of relative binding favorability without relying on undefined qualitative categories.

The combined results of composition and docking analyses suggest that Indian mustard oil could be the most plausible source of cholinesterase-active fatty acids at the single-compound level. This oil contains α-linolenic acid, erucic acid, linoleic acid, gondoic acid, and nervonic acid, several of which ranked among the best ligands in the AChE and BChE models. Passion fruit oil could also be relevant, particularly given its high linoleic acid content and the presence of nervonic acid, which may help explain its strong experimental cholinesterase-inhibitory profile. However, the biological activity of the whole oil cannot be attributed exclusively to the docked fatty acids, since the wet-lab inhibition values likely reflect not only the major lipids but also the contribution of minor constituents and matrix effects. This is particularly important because passion fruit oil showed the highest experimental BChE inhibition, whereas Indian mustard oil was compositionally richer in some of the top-ranked ligands. Accordingly, docking should be interpreted here as a prioritization tool rather than direct proof of causality. Tyrosinase displayed a different pattern. Although α-linolenic acid and linoleic acid again ranked highest among the fatty acids, their affinities were clearly lower than those observed in cholinesterases, and the best poses were split between the dicopper catalytic region and an adjacent kojic-acid-like pocket. This suggests a weaker or more indirect interaction mode, likely dominated by hydrophobic accommodation rather than the stronger polar or metal-centered recognition typically expected for classical aromatic tyrosinase inhibitors. This reading agrees with the experimental data, in which tyrosinase inhibition was observed for avocado and Indian mustard oils but not for passion fruit oil, indicating that whole-oil behavior may depend on compositional balance rather than on a single major fatty acid. Overall, α-linolenic acid seemed the most robust docking candidate because it ranked among the best ligands in both AChE models, in BChE, and in both tyrosinase regions, while maintaining acceptable variability across triplicate runs. Linoleic acid and nervonic acid also showed consistent performance, particularly in cholinesterases. These findings corroborate the hypothesis that the multifunctional biological properties of the investigated oils are shaped, at least in part, by their specific unsaturated fatty-acid signatures, with Indian mustard oil showing the strongest compositional enrichment in candidate inhibitory fatty acids, passion fruit oil showing a composition compatible with strong cholinesterase-related effects, and avocado oil providing a more selective oleic-acid-dominated profile. The combined results of composition and docking analyses suggest that Indian mustard oil could be the most plausible source of cholinesterase-active fatty acids at the single-compound level. This oil contains α-linolenic acid, erucic acid, linoleic acid, gondoic acid, and nervonic acid, several of which ranked among the best ligands in the AChE and BChE models. Passion fruit oil could also be relevant, particularly given its high linoleic acid content and the presence of nervonic acid, which may help explain its strong experimental cholinesterase-inhibitory profile. However, the biological activity of the whole oil cannot be attributed exclusively to the docked fatty acids, since the wet-lab inhibition values likely reflect not only the major lipids but also the contribution of minor constituents and matrix effects. This is particularly important because passion fruit oil showed the highest experimental BChE inhibition, whereas Indian mustard oil was compositionally richer in some of the top-ranked ligands. Accordingly, docking should be interpreted here as a prioritization tool rather than direct proof of causality. Representative 3D poses and 2D protein-ligand interaction diagrams are provided as Supplementary Figures 1–3 to show ligand orientation within the enzymatic binding regions and to clarify whether recurrent contacts from Table 3 occur in catalytic, gorge-defining, peripheral, or adjacent binding regions. Tyrosinase displayed a different pattern. Although α-linolenic acid and linoleic acid again ranked highest among the fatty acids, their affinities were clearly lower than those observed in cholinesterases, and the best poses were split between the dicopper catalytic region and an adjacent kojic-acid-like pocket. This suggests a weaker or more indirect interaction mode, likely dominated by hydrophobic accommodation rather than the stronger polar or metal-centered recognition typically expected for classical aromatic tyrosinase inhibitors. This reading agrees with the experimental data, in which tyrosinase inhibition was observed for avocado and Indian mustard oils but not for passion fruit oil, indicating that whole-oil behavior may depend on compositional balance rather than on a single major fatty acid. Overall, α-linolenic acid seemed the most robust docking candidate because it ranked among the best ligands in both AChE models, in BChE, and in both tyrosinase regions, while maintaining acceptable variability across triplicate runs. Linoleic acid and nervonic acid also showed consistent performance, particularly in cholinesterases. These findings corroborate the hypothesis that the multifunctional biological properties of the investigated oils are shaped, at least in part, by their specific unsaturated fatty-acid signatures, with Indian mustard oil showing the strongest compositional enrichment in candidate inhibitory fatty acids, passion fruit oil showing a composition compatible with strong cholinesterase-related effects, and avocado oil providing a more selective oleic-acid-dominated profile.

### 3.6 Correlation between fatty acid composition and biological activities of avocado, Indian mustard, and passion fruit seed oils, and their potential application

The correlation analysis suggested a potential association between the fatty acid profiles of the three seed oils and biological activity. Several fatty acids, including linoleic acid, eicosenoic acid, behenic acid, and lignoceric acid, demonstrated significant correlations with the antioxidant activity of seed oils (*r* = −0.90, 0.96, −0.87, and –0.86, respectively). The antioxidant effect measured by the ABTS assay was robustly correlated with linolenic acid (*r* = 0.98) and erucic acid (*r* = 0.98). Antiarthritic activity was negatively correlated with linolenic acid (*r* = −0.96) and gondoic acid (*r* = −0.95).

Regarding neuroprotective capacity, the acetylcholinesterase (AChE) inhibitory effect was highly correlated with linoleic acid, gondoic acid, and erucic acid (*r* = 0.99 for each). The butyrylcholinesterase (BChE) inhibitory effect was correlated with stearic acid (*r* = 0.94), elaidic acid (*r* = 0.87), and, to a lesser extent, linoleic acid (*r* = 0.74). Linoleic acid (*r* = −0.99), elaidic acid (*r* = −0.98), and eicosenoic acid (*r* = −0.97) also demonstrated strong negative correlations with tyrosinase inhibition. The negative correlations observed between linoleic acid content and DPPH IC_50_ (*r* = −0.98), as well as tyrosinase inhibition (*r* = −0.93), are consistent with the known membrane-interacting and radical-scavenging properties of polyunsaturated fatty acids (57, 85, 86). Indian mustard oil, rich in erucic and linolenic acids, displayed anti-arthritic and antioxidant activity. Correlation analysis suggested that higher levels of long-chain UFAs were associated with increased anti-arthritic and antioxidant activity (the latter evaluated using the DPPH assay), consistent with previously reported anti-inflammatory and biofilm-modulating properties of these compounds (87, 88). Avocado oil, dominated by oleic acid (92.4%), exhibited a moderate activity, except for the AChE inhibition, where such fatty acid demonstrated a r value not exceeding 0.45.

**FIGURE 2:**
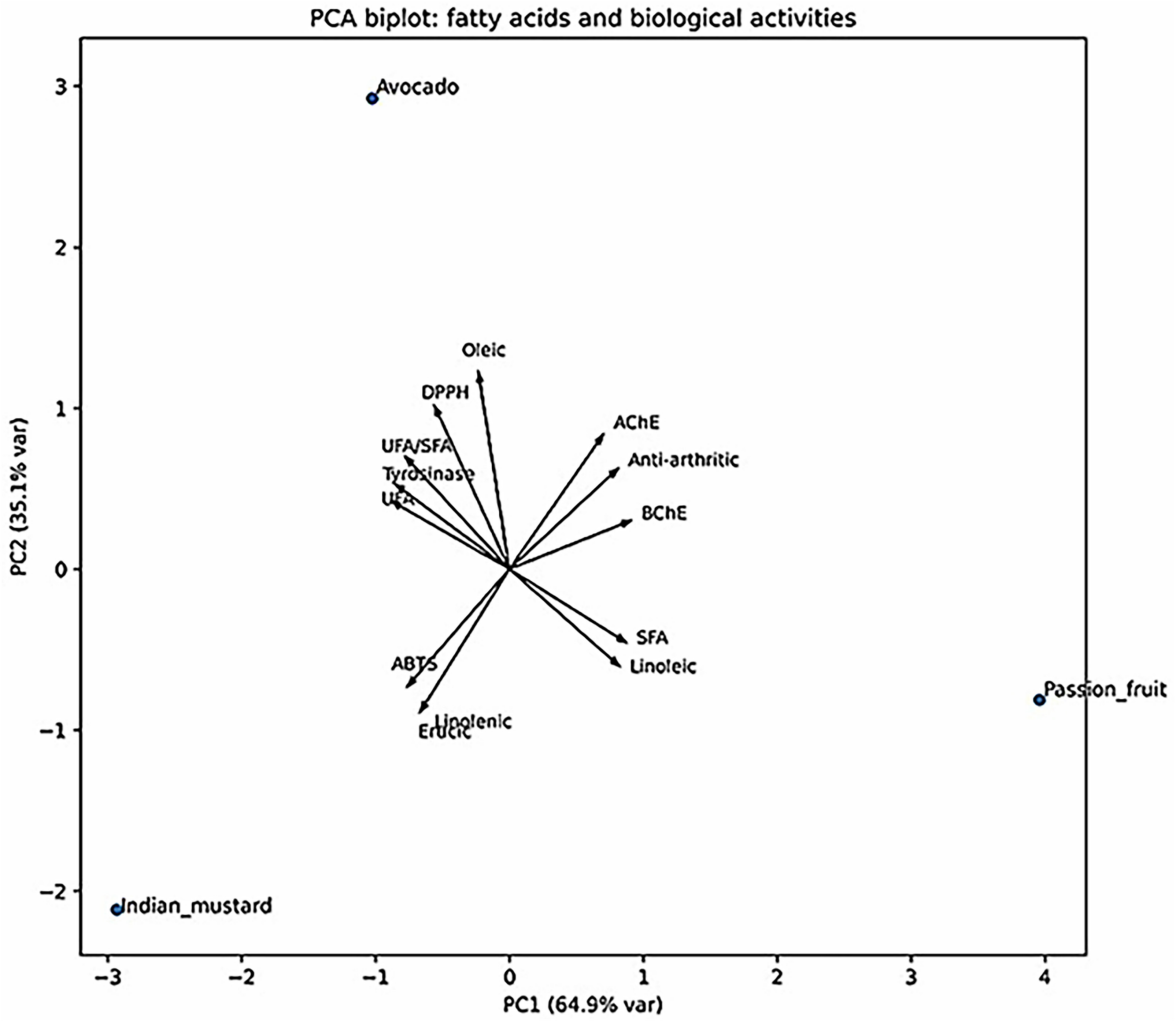
Principal component analysis (PCA) biplot showing the relationships between fatty acid composition and biological activities of the investigated seed oils. Oil samples are represented as score points, while variables are displayed as loading vectors. The variables included fatty acid profile parameters (e.g., oleic acid, linoleic acid, linolenic acid, erucic acid, total saturated fatty acids, total unsaturated fatty acids, and the unsaturated/saturated fatty acid ratio) together with biological activity parameters such as antioxidant activity (DPPH IC_50_ and ABTS values), anti-arthritic activity, acetylcholinesterase (AChE) inhibition, butyrylcholinesterase (BChE) inhibition, and tyrosinase inhibitory activity. The direction and length of the vectors indicate each variable’s contribution to the principal components and highlight potential associations between fatty acid composition and biological properties. PC1 and PC2 explained 64.9 and 35.1% of the variance, respectively.

To better understand the relationships between the chemical composition of the oils and their biological effects, two complementary principal component analyses (PCA) were performed. Multivariate analysis was used as an exploratory tool to integrate compositional and functional datasets and to identify possible structure–activity relationships among the investigated oils. Prior to PCA, all variables were standardized (z-score normalization) to remove scale differences among the measured parameters and ensure equal weighting of chemical and biological variables. PCA was then applied to the standardized dataset using a covariance-based approach, and the first two principal components (PC1 and PC2) were retained for visualization and interpretation. The PCA integrated fatty acid composition with antioxidant, anti-arthritic, and enzyme inhibitory activities (Figure 2). The analysis revealed a clear separation among the three oils based on their chemical composition and biological effects. Avocado oil was aligned with oleic acid and showed a closer association with acetylcholinesterase (AChE) inhibitory activity. Indian mustard oil clustered separately and was primarily associated with erucic acid, a major component of its lipid profile. In contrast, passion fruit oil was distributed along the linoleic acid axis and showed stronger associations with butyrylcholinesterase (BChE) inhibitory activity. Overall, the PCA results suggest that differences in fatty acid composition contribute to the variability in the biological activities observed among the investigated oils. In particular, the degree of unsaturation and the presence of specific fatty acids appear to influence antioxidant and enzyme inhibitory properties.

### 3.7 Antibiofilm activity

Beyond enzymatic and antioxidant activities, the antimicrobial potential of the oils was further explored by assessing their ability to inhibit bacterial biofilm formation and metabolic activity. Our study evaluated the antibiofilm activity of avocado, Indian mustard, and passion fruit seed oils against six clinically and industrially relevant bacterial pathogens: *Acinetobacter baumannii*, *Escherichia coli*, *Klebsiella pneumoniae*, *Listeria monocytogenes*, *Pseudomonas aeruginosa*, and *Staphylococcus aureus*. The work was performed after the minimum inhibitory concentration was evaluated (Table 4).

**TABLE 4:** Minimal inhibitory concentration of avocado, Indian mustard, and passion fruit seed oil (μL/mL).

Minimal inhibitory concentration						
Seed oil	AB	EC	KP	LM	PA	SA
Avocado	35.00^a^ ± 2.00	34.00^a^ ± 1.00	> 50.00^b^	33.00^a^ ± 1.00	34.00^a^ ± 1.00	32.00^a^ ± 1.00
Indian mustard	34.00^a^ ± 1.00	34.00^a^ ± 1.00	40.00^b^ ± 2.00	33.00^a^ ± 1.00	> 50.00^b^	32.00^a^ ± 1.00
Passion fruit	34.00 ± 1.00	34.00^a^ ± 1.00	> 50.00^b^	33.00^a^ ± 1.00	40.00^b^ ± 2.00	40.00^b^ ± 2.00
Control	32.00 ± 1.00	30.00 ± 1.00	32.00 ± 1.00	30.00 ± 1.00	30.00 ± 1.00	28.00 ± 1.00

Values are means ± SD of three measurements, means within each column with different letters (a–b) differ significantly (*p* ≤ 0.05). AB, *Acinetobacter baumannii*; EC, *Escherichia coli*; KP, *Klebsiella pneumoniae*; LM, *Listeria monocytogenes*; PA, *Pseudomonas aeruginosa*; SA, *Staphylococcus aureus*. ANOVA followed by Dunnett’s multiple comparison test. *p* < 0.05.

Two complementary methodologies were employed: crystal violet (CV) staining to quantify total biofilm biomass and the MTT assay to assess the metabolic activity of viable sessile cells. The oils were introduced at two concentrations (10 and 20 μL/mL) at two critical time points: at the onset of bacterial growth (T0, representing immature biofilm) and after 24 h of biofilm maturation (T24, representing mature biofilm). This approach enabled evaluation of the comparative efficacy of the three oils and clarification of their distinct modes of action and temporal dynamics against various bacterial species and biofilm developmental stages. Results are presented in Tables 5 (crystal violet assay) and 6 (MTT test).

**TABLE 5:** Effect of the oils (tested at 10 and 20 mL/mL) on biofilm adhesion (indicated as CV0) and mature biofilm (indicated as CV24).

CV0	Avocado		Indian mustard		Passion fruit	
AB	10 mL/mL	20 mL/mL	10 mL/mL	20 mL/mL	10 mL/mL	20 mL/mL
AB	0.00 ± 0.00	15.66^a^ ± 0.98	0.00 ± 0.00	0.00 ± 0.00	0.00 ± 0.00	21.14^a^ ± 1.76
EC	0.00 ± 0.00	0.00 ± 0.00	0.00 ± 0.00	20.95^a^ ± 1.55	0.00 ± 0.00	14.54^a^ ± 1.12
KP	16.94^a^ ± 1.67	31.98^b^ ± 2.67	3.11^a^ ± 0.12	29.96^b^ ± 2.02	15.40^a^ ± 1.02	39.54^b^ ± 2.76
LM	0.00 ± 0.00	2.47^a^ ± 0.06	0.00 ± 0.00	5.81^a^ ± 0.12	0.00 ± 0.00	28.44^b^ ± 1.12
PA	22.46^a^ ± 1.13	27.51^b^ ± 1.69	0.00 ± 0.00	21.74^a^ ± 1.22	46.88^b^ ± 1.08	73.10^c^ ± 1.78
SA	0.00 ± 0.00	0.00 ± 0.00	0.00 ± 0.00	0.00 ± 0.00	0.00 ± 0.00	0.75^nd^ ± 0.02
**CV24**	**Avocado**		**Indian mustard**		**Passion fruit**	
	**10 mL/mL**	**20 mL/mL**	**10 mL/mL**	**20 mL/mL**	**10 mL/mL**	**20 mL/mL**
AB	14.30^a^ ± 1.12	29.41^b^ ± 1.54	0.00 ± 0.00	32.69^b^ ± 2.02	0.00 ± 0.00	0.00 ± 0.00
EC	0.00 ± 0.00	28.04^b^ ± 1.71	21.79^a^ ± 1.13	50.04^b^ ± 3.45	18.45^a^ ± 1.12	41.96^b^ ± 2.17
KP	16.35^a^ ± 1.67	43.34^b^ ± 3.22	38.75^b^ ± 1.22	42.67^b^ ± 3.02	0.00 ± 0.00	37.39^b^ ± 1.65
LM	58.22^c^ ± 3.09	77.80^c^ ± 3.44	65.49^c^ ± 1.41	66.28^c^ ± 1.14	40.33^b^ ± 1.11	48.16^b^ ± 1.54
PA	58.09^c^ ± 3.64	68.76^c^ ± 2.08	35.04^b^ ± 2.36	63.33^c^ ± 1.26	5.78^a^ ± 0.08	25.47^a^ ± 1.08
SA	26.54^b^ ± 1.57	33.67^b^ ± 2.13	45.99^b^ ± 2.87	68.46^c^ ± 1.69	29.01^b^ ± 1.02	35.72^b^ ± 2.32

Results were compared with the control (untreated bacteria), for which inhibition = 0 was assumed. Results are expressed as percentages and represent the average of three independent experiments ± SD. Different superscript letters within the same row indicate significant differences among treatments for the same bacterial strain according to one-way ANOVA followed by Tukey’s test (*p* < 0.05). Statistical comparisons were not performed between different bacterial strains. AB, *Acinetobacter baumannii*; EC, *Escherichia coli*; KP, *Klebsiella pneumoniae*; LM, *Listeria monocytogenes*; PA, *Pseudomonas aeruginosa*; SA, *Staphylococcus aureus*.

#### 3.7.1 Crystal violet assay

The three oils demonstrated distinct antibiofilm activity profiles, both in overall efficacy and in strain-specific susceptibility patterns. Table 5 shows the effect of the oils on immature biofilm.

Passion fruit seed oil exhibited the highest inhibitory activity against *P. aeruginosa* at the immature biofilm stage (T0), achieving 73.10% ± 1.78 inhibition at 20 μL/mL. This result represents the most substantial single-strain inhibition among the three oils at T0. The pronounced effect against *P. aeruginosa*, a well-established biofilm-forming opportunistic pathogen (89), is particularly significant. Interestingly, pronounced inhibition was observed during the early stages of *P. aeruginosa* biofilm formation, particularly in the CV0 assay. This suggests that the oils may interfere with initial adhesion processes or early biofilm development rather than disrupt mature biofilm structures. Passion fruit seed oil also showed notable activity against immature biofilms of *K. pneumoniae* (39.54% ± 2.76) and *L. monocytogenes* (28.44% ± 1.12). However, its efficacy declined at T24, with inhibition of *P. aeruginosa* decreasing to 25.47% ± 1.08 and *A. baumannii* exhibiting complete resistance. In contrast, inhibitory activity increased against *L. monocytogenes* (from 28.44 ± 1.12 to 48.16% ± 1.54) and, most notably, against *S. aureus* biofilm, with a marked increase from 0.75 ± 0.02 to 35.72% ± 2.32. Avocado oil demonstrated an opposite temporal trend, with antibiofilm activity generally increasing from T0 to T24. At the mature biofilm stage, avocado oil achieved the highest inhibition against *L. monocytogenes* (77.80% ± 3.44 at 20 μL/mL), followed by *P. aeruginosa* (68.76% ± 2.08) and *K. pneumoniae* (43.34% ± 3.22). Its activity was also considerable against *E. coli* and *S. aureus*, increasing from 0 to 28.04% ± 1.71 and 33.67% ± 2.13, respectively, at 20 μL/mL. The greater susceptibility of *L. monocytogenes* could be related to the absence of an outer membrane, which facilitates interactions between hydrophobic lipid compounds and the bacterial cell envelope. This time-dependent increase suggests that avocado oil might disrupt biofilm maturation and extracellular polymeric substance (EPS) production. The strong inhibition of *L. monocytogenes* is particularly relevant to food-industry applications (90). Indian mustard oil displayed distinct temporal dynamics, with negligible activity at T0 but a substantial reduction in mature biofilm. At T24, Indian mustard oil demonstrated strong inhibitory activity against *S. aureus* (68.46% ± 1.69), *L. monocytogenes* (66.28% ± 1.14), *P. aeruginosa* (63.33% ± 1.26), and *E. coli* (50.04% ± 3.45). This delayed-action profile suggests mechanisms involving gradual accumulation within the biofilm matrix or progressive interference with bacterial signaling systems.

#### 3.7.2 MTT assay

The MTT assay measures the metabolism of sessile cells by quantifying the reduction of tetrazolium salts by NAD(P)H-dependent dehydrogenases. Results are shown in Table 6 and refer to the action of the oils on sessile cells within the immature and mature biofilm (indicated as MTT0 and MTT24, respectively).

**TABLE 6:** Inhibitory activity (calculated as percentage, with respect to the untreated cells) of the three seed oil (tested at 10 and 20 mL/mL) on sessile cells metabolism within the immature and mature biofilm (indicated as MTT0 and MTT24, respectively).

MTT0	Avocado		Indian mustard		Passion fruit	
	10 mL/mL	20 mL/mL	10 mL/mL	20 mL/mL	10 mL/mL	20 mL/mL
AB	29.06^b^ ± 1.63	36.71^b^ ± 2.87	12.27^a^ ± 0.88	23.96^a^ ± 1.03	0.00 ± 0.00	23.29^a^ ± 1.02
EC	23.91^a^ ± 1.13	43.36^b^ ± 3.34	0.00 ± 0.00	24.20^a^ ± 2.02	0.00 ± 0.00	23.36^a^ ± 1.04
KP	9.80^a^ ± 0.12	41.50^b^ ± 2.25	0.00 ± 0.00	0.00 ± 0.00	0.00 ± 0.00	15.58^a^ ± 1.11
LM	29.57^b^ ± 2.02	31.39^b^ ± 1.67	0.00 ± 0.00	7.34^a^ ± 0.57	0.00 ± 0.00	22.42^a^ ± 2.06
PA	17.23^a^ ± 1.12	41.67^b^ ± 1.88	22.11^a^ ± 1.05	39.77^b^ ± 2.76	0.00 ± 0.00	5.64^a^ ± 0.04
SA	17.43^a^ ± 1.11	36.19^b^ ± 2.04	0.00 ± 0.00	0.00 ± 0.00	0.00 ± 0.00	5.67^a^ ± 0.11
**MTT24**	**Avocado**		**Indian mustard**		**Passion fruit**	
	**10 mL/mL**	**20 mL/mL**	**10 mL/mL**	**20 mL/mL**	**10 mL/mL**	**20 mL/mL**
AB	16.83^a^ ± 1.41	19.46^a^ ± 1.57	0.00 ± 0.00	32.70^b^ ± 3.30	0.00 ± 0.00	0.00 ± 0.00
EC	41.74^b^ ± 2.14	53.63^c^ ± 1.88	21.74^a^ ± 1.03	30.01^b^ ± 2.76	0.00 ± 0.00	0.00 ± 0.00
KP	9.08^a^ ± 0.27	19.99^a^ ± 1.45	16.69^a^ ± 1.61	26.52^b^ ± 1.06	0.00 ± 0.00	0.00 ± 0.00
LM	0.00 ± 0.00	33.14^b^ ± 3.44	2.88^a^ ± 0.03	18.01^a^ ± 1.67	4.56^a^ ± 0.03	13.0^a^ ± 1.08
PA	36.88^b^ ± 3.08	47.93^b^ ± 3.11	20.67^a^ ± 1.05	34.30^b^ ± 2.12	0.00 ± 0.00	7.14^a^ ± 0.11
SA	0.00 ± 0.00	0.00 ± 0.00	0.00 ± 0.00	0.00 ± 0.00	0.00 ± 0.00	0.00 ± 0.00

Results were compared with the control (untreated bacteria), for which inhibition was assumed to be 0. Results are expressed as percentages and represent the average of three independent experiments ± SD. Different superscript letters within the same row indicate significant differences among treatments for the same bacterial strain according to one-way ANOVA followed by Tukey’s test (*p* < 0.05). Statistical comparisons were not performed between different bacterial strains. AB, *Acinetobacter baumannii*; EC, *Escherichia coli*; KP, *Klebsiella pneumoniae*; LM, *Listeria monocytogenes*; PA, *Pseudomonas aeruginosa*; SA, *Staphylococcus aureus*.

Metabolic activity analysis revealed distinct mechanistic profiles among the tested oils. Passion fruit oil typically resulted in low MTT inhibition despite substantial reductions in biomass, suggesting a primary effect on biofilm structure and extracellular polymeric substances rather than on cellular viability. The most pronounced effect was observed with *P. aeruginosa*, which exhibited 73.10% ± 1.78 CV0 inhibition compared to only 5.64% ± 0.04 MTT0 inhibition. Avocado oil demonstrated greater metabolic inhibition, particularly at T0, with significant effects against *E. coli* (43.36% 3.34 ± ), *K. pneumoniae* (41.50% ± 2.25), and *P. aeruginosa* (41.67% ± 1.88). At T24, *E. coli* exhibited the highest inhibition (53.63% ± 1.88), followed by *P. aeruginosa* (47.93% ± 3.11). These findings suggest that avocado oil could exert dual effects, by disrupting both biofilm architecture and the metabolic activity of embedded cells. Indian mustard oil produced minimal metabolic inhibition at T0 but showed increased inhibition at T24, with the highest effects against *P. aeruginosa* (34.30% ± 2.12), *A. baumannii* (32.70% ± 3.30), and *E. coli* (30.01% ± 2.76). *S. aureus* exhibited a 68.46% ± 1.69 reduction in biomass but 0% metabolic inhibition, indicating a purely structural mode of disruption.

Differences between CV and MTT assays suggest that oils affect both biofilm biomass and metabolic activity, possibly acting at different stages of biofilm development. The greater inhibition observed after prolonged exposure—especially in mature biofilms—points to a time-dependent effect, likely due to the gradual penetration of lipids into the biofilm matrix. Combined CV and MTT results further suggest hypothetic different modes of antibiofilm action:

- Matrix-Targeting (CV ≫ MTT): Oils mainly disrupt the biofilm structure or prevent bacterial adhesion (91, 92). For example, passion fruit oil applied to *P. aeruginosa* at T0 reduced biomass by 73.10% ± 1.78 (CV) but had minimal effect on metabolism (5.64% ± 0.04 MTT). Indian mustard oil on *S. aureus* at T24 showed a similar pattern. These effects may sensitize bacteria to antibiotics without promoting resistance (93).
- Metabolic-Targeting (MTT ≥ CV): Oils primarily could inhibit the metabolism of sessile cells rather than reducing biomass. Avocado oil on *E. coli* at T0 did not lower biomass (0% CV) but reduced metabolic activity by 43.36% ± 3.34 (MTT), likely by disrupting bacterial membranes (83). The observed discrepancies between CV and MTT may also indicate impaired exopolysaccharide production (94).
- Temporal Shifts: Some strains, such as *S. aureus*, became more susceptible at T24, with Gram-positive bacteria generally more affected at this later point (95). Nevertheless, certain oils were still able to inhibit Gram-negative bacteria, probably through membrane disruption (96).

To assess the potential relationship between the lipid composition of the tested oils and their antibiofilm activity, Pearson correlation analysis of fatty acid composition and antibiofilm parameters, calculated for each experimental condition (immature and mature biofilm) at the highest concentration (20 μL/mL) used in the antibiofilm activity tests, suggested a strong association between lipid unsaturation and antibiofilm efficacy. To improve clarity, results could be grouped by biofilm maturity and bacterial Gram type, thereby highlighting major patterns and reducing cognitive load. First, when considering immature biofilms, total unsaturated fatty acids (UFAs) and the unsaturated/saturated ratio (UFAs/SFAs) exhibited positive correlations with metabolic inhibition (*r* = 0.74–1.00). Within this group, oleic acid exerted a notably inhibitory effect on immature biofilms of *A. baumannii* (*r* = 0.97) and *K. pneumoniae* (*r* = 0.83). In contrast, negative correlations were observed for *E. coli*, *L. monocytogenes*, and *P. aeruginosa* (*r* = −0.70, −0.72, and −0.68, respectively), suggesting species-specific responses. The presence of linoleic acid was particularly effective in inhibiting immature biofilms of *E. coli* (*r* = 0.96), *L. monocytogenes* (*r* = 0.94), and *P. aeruginosa* (*r* = 0.98). Interestingly, total SFAs positively influenced the inhibition of immature biofilms of *E. coli* (*r* = 0.90), *L. monocytogenes* (*r* = 0.88), and *P. aeruginosa* (*r* = 0.93), while UFAs remained more effective against *A. baumannii* (*r* = 0.92). Next, focusing on mature biofilms, oleic acid again contributed to inhibiting *A. baumannii* (*r* = 0.88), *L. monocytogenes* (*r* = 0.82), and *P. aeruginosa* (*r* = 0.87). However, total UFAs did not consistently enhance inhibition in mature *E. coli* and *S. aureus* biofilms; instead, they were associated with either no improvement or even negative correlations (*r* = −0.72 and *r* = 0.67, respectively), suggesting alternative or compensatory mechanisms at this stage. Grouping these findings by biofilm maturity and Gram type reveals a clearer picture of the complex relationships between fatty acid profiles and biofilm inhibition. Gram-negative bacteria (*E. coli*, *A. baumannii*, *K. pneumoniae*, *P. aeruginosa*) show varying degrees of sensitivity to specific fatty acids, with Gram-positive *L. monocytogenes* displaying strong positive correlations to both oleic and linoleic acid inhibition, especially in immature and mature biofilms alike. *S. aureus*, another Gram-positive, exhibited generally weaker or nonsignificant correlations for most fatty acids across parameters. The links between unsaturation and metabolic inhibition support a mechanistic hypothesis where targeting membrane composition could serve as an actionable strategy to impair biofilm function. These data suggest a broad-spectrum involvement of polyunsaturated fatty acids in impairing both biofilm stability and bacterial cell viability, possibly through disruption of membrane integrity and interference with metabolic pathways (85). However, the moderate fraction of saturated fatty acids in passion fruit oil may partially explain its reduced activity against mature biofilms, consistent with the negative correlation between SFA content and antibiofilm performance. This underscores how the balance between UFAs and SFAs can significantly influence the bio-efficacy of the oils, depending on the target (e.g., mature vs. immature biofilms) (87, 88). The results provide a framework for the targeted selection of seed oils based on the application context. Compared with synthetic antimicrobials, these oils could offer potential advantages, including consumer acceptance, multi-target activity, and compatibility with conventional agents. Future work will focus on standardizing the composition, optimizing the formulation, and evaluating safe parameters, also through *in vivo* and application-based studies (37, 93).

### 3.8 Effect of the presence of avocado, Indian mustard, and passion fruit seed oil on the probiotic growth

Table 7 shows the effect of the three seed oils on probiotic growth.

**TABLE 7:** Effect of the presence of the avocado, Indian mustard, and passion fruit seed oil on the probiotic growth.

Strain	Avocado	Indian mustard	Passion fruit	Control
*L. plantarum*	0.24 ± 0.05^***^	0.13 ± 0.01^ns^	0.36 ± 0.07 ^***^	0.10 ± 0.04
*L. paracasei*	0.22 ± 0.07^**^	0.19 ± 0.03^**^	0.19 ± 0.06^**^	0.16 ± 0.04
*L. bulgaricus*	1.72 ± 0.07^***^	1.64 ± 0.04^***^	1.35 ± 0.06 *	1.38 ± 0.01
*L. gasseri*	0.21 ± 0.07^**^	0.12 ± 0.02^ns^	0.14 ± 0.01^***^	0.12 ± 0.01

Values are expressed as mean OD 600 ± SD (*n* = 3). Statistical differences between treatments and the control were evaluated using one-way ANOVA followed by Dunnett-type multiple comparison. Significance levels:

*p < 0.05;

**p < 0.01;

***p < 0.001; ns, not significant.

The growth of the four lactic acid bacteria strains showed clear differences depending on the oil used as a supplement in the culture medium, highlighting a strong strain-dependent response to the lipid environment. Overall, avocado oil induced the most consistent increase in bacterial growth, whereas Indian mustard and passion fruit oils produced more variable responses across strains. These differences are likely associated with the lipid characteristics of the oils, particularly their degree of fatty acid unsaturation, which may influence membrane fluidity and bacterial metabolic activity. Among the tested strains, *L. bulgaricus* exhibited the highest absolute growth values across all treatments, confirming its robust metabolic activity and high tolerance to lipid supplementation. Growth increased in the presence of avocado oil and remained relatively high with mustard oil, while slightly lower values were observed with passion fruit oil. *L. plantarum* showed the most pronounced response to oil supplementation. In particular, the presence of passion fruit oil led to a marked increase in growth compared with the control, while avocado oil also exerted a strong stimulatory effect. This behavior is consistent with the well-known metabolic versatility of *L. plantarum*, which is frequently found in plant-derived environments and exhibits a high capacity to adapt to lipid-rich substrates. *L. paracasei* exhibited a more moderate response to the treatments, with only slight variations among the oils. Avocado oil produced the highest growth values, whereas mustard and passion fruit oils resulted in growth levels closer to the control. Finally, *L. gasseri* exhibited moderate stimulation, primarily in the presence of avocado oil, whereas growth in the other treatments remained comparable to the control. This suggests a more limited capacity of this strain to benefit from lipid supplementation. With respect to the fatty acid composition, we observed that the growth of LAB could be correlated to the overall degree of lipid unsaturation of the oils. Avocado oil, which showed the strongest growth-promoting effect across most strains, has the highest unsaturated-to-saturated fatty acid ratio. Oils with a high degree of unsaturation might improve the physicochemical properties of bacterial membranes, facilitate nutrient transport, and enhance the activity of membrane-associated enzymes. In contrast, oils with lower levels of unsaturation may exert less pronounced stimulatory effects on bacterial growth. A positive trend was observed between the unsaturated/saturated fatty acid ratio of the oils and the mean growth of *Lactobacillus* strains. Despite the limited number of data points, the observed trend suggests that oils richer in unsaturated fatty acids may favor LAB growth. Furthermore, although the present study did not investigate the fatty acid composition of bacterial membranes after growth with different oils, previous studies have demonstrated that lactobacilli can incorporate exogenous unsaturated fatty acids into their membrane lipids, thereby influencing membrane fluidity and stress tolerance. Therefore, Partanen et al. (97) observed that the oils containing the lowest amounts of saturated long-chain fatty acids promoted the growth of *L. delbrueckii* most effectively in the presence of saturated fatty acids; Kankaanpaa et al. (98) demonstrated that PUFA supplementation to the growth medium of LAB determined a positive influence on the bacterial growth, and suggested that such acids could interfere with microbial adhesion to the intestinal surfaces. The results insinuate once again that plant-derived oils can modulate the growth of technologically relevant lactic acid bacteria and may therefore influence fermentation performance or probiotic viability in lipid-containing food matrices. Oils with higher levels of unsaturation seem to provide more favorable conditions for bacterial growth, whereas oils with different lipid structures may produce strain-dependent effects. The prebiotic index (P.I.) (99) applied to fatty acids highlighted that growth of *L. plantarum* was most positively affected by the presence of all oils: passion fruit oil (P.I. = 3.34), avocado oil (P.I. = 2.25), and Indian mustard oil (P.I. = 1.15). *L. paracasei* growth seemed positively affected, although in a less marked manner, by the presence of avocado, Indian mustard, and passion fruit oils, with P.I. values of 1.38, 1.20, and 1.15, respectively. *L. bulgaricus* obtained a positive influence by the presence of avocado (P.I. = 1.24) and Indian mustard (P.I. = 1.18), but not much when we added passion fruit oil, whose presence did not cause any evident increase in its growth (P.I. = 0.97). The growth of *L. gasseri* seemed positively affected by the presence of avocado oil (P.I. = 1.79) and less by passion fruit, which determined a P.I. = 1.16; on the other hand, its growth did not seem to be affected by the presence of Indian mustard oil (P.I. = 0.99).

**FIGURE 3:**
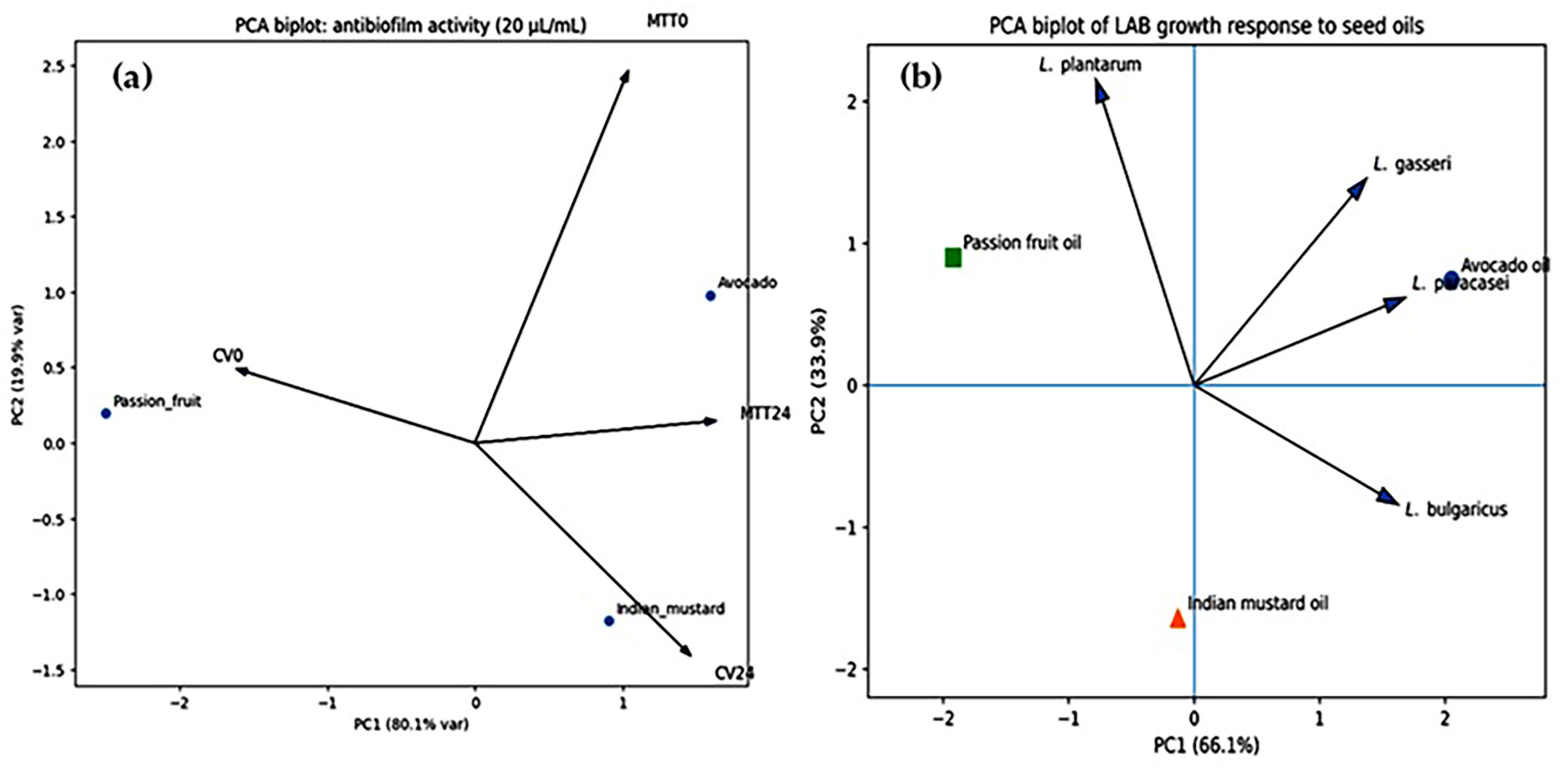
**(a)** Principal component analysis (PCA) biplot of antibiofilm activity parameters of the investigated oils. The analysis was performed using metabolic activity (MTT0 and MTT24) and biofilm biomass measurements (CV0 and CV24) obtained at 20 μL/mL against the tested bacterial strains. The first two principal components explained 80.1 and 19.9% of the total variance, respectively, highlighting differences in metabolic inhibition and biofilm biomass reduction among the investigated oils. **(b)** Principal component analysis (PCA) biplot showing the relationship between the seed oils investigated and the growth response of lactic acid bacteria. PC1 and PC2 explained 66.1 and 33.9% of the total variance, respectively. Oils are represented as score points, while antibiofilm variables and growth are shown as loading vectors.

To further explore the relationships between the oils, their antibiofilm activity, and LAB-growth-stimulating activity, two principal component analyses were performed (Figures 3a,b, respectively. The principal component analysis (PCA) biplot, constructed using antibiofilm activity parameters (MTT0, MTT24, CV0, and CV24 at 20 μL/mL, Figure 3a) and the growth of the LAB strains at 20 μL/mL (Figures 3b), demonstrated clear differentiation among the three seed oils examined.

These two PCA biplots provided a reliable representation of the overall microbiological differences among the oils. In both plots, PC1 mainly separated avocado oil from passion fruit oil. Avocado oil was located on the positive side of PC1, whereas passion fruit oil was positioned on the negative side. Avocado oil, which had the highest proportion of oleic acid and the highest unsaturated-to-saturated fatty acids ratio, was in the PCA space associated with antibiofilm activity and with the growth of *L. gasseri*, *L. paracasei*, and *L. bulgaricus*. This distribution indicates that avocado oil was associated with a microbiological profile characterized by coexisting antibiofilm activity and good compatibility with selected LAB strains. In contrast, the negative position of passion fruit oil along PC1 suggests a microbiological behavior less associated with these variables and therefore distinct from that of avocado oil. Passion fruit oil, mainly characterized by polyunsaturated fatty acids such as linoleic acid and elaidic acid, exhibited a microbiological profile distinct from that of avocado oil. Polyunsaturated fatty acids are known to interact strongly with bacterial membranes and may influence membrane permeability and oxidative processes, which may partly explain the unique clustering of passion fruit oil in the multivariate space (83). Indian mustard oil, containing long-chain fatty acids such as erucic acid and linolenic acid, exhibited distinct behavior, occupying a specific region of the PCA space. The position of this oil along the second principal component (PC2) suggests that these fatty acids may confer more selective antimicrobial activity, particularly against certain pathogenic strains, while exerting a less pronounced effect on lactic acid bacterial growth. Overall, microbiological PCA supports the hypothesis that differences in fatty acid composition may simultaneously modulate both antimicrobial activity and LAB growth. This result might differ not only in their inhibitory action against undesirable microorganisms but also in their compatibility with technologically and functionally relevant bacterial species. Because the PCA was performed on a limited number of oil samples, the multivariate distribution should be interpreted primarily as an exploratory representation of microbiological trends rather than as definitive evidence of causality. Nevertheless, the clear separation of the oils in the PCA space indicates that their microbiological behavior is sufficiently distinct to justify further investigation of the relationship between lipid composition and functional microbial responses.

The biofilm-inhibitory effects of avocado, Indian mustard, and passion fruit seed oils against pathogenic strains, along with their capacity to promote the growth of four probiotic strains, are of particular scientific interest, mainly because certain pathogenic strains are implicated in the mechanisms underlying the onset of inflammatory (100), and neurodegenerative diseases (101, 102). These oils may exert direct effects on disease processes or on the pathogens that contribute to disease initiation. Additionally, they may stimulate the growth of probiotic bacteria, which can act directly on disease (103, 104) or inhibit the proliferation of pathogenic bacteria, thereby indirectly preventing and limiting neurodegenerative and inflammatory diseases (42).

## 4 Conclusion

This comparative study demonstrates that avocado, Indian mustard, and passion fruit seed oils possess distinct fatty acid profiles, each associated with specific biological and functional properties. Passion fruit oil, with a high proportion of polyunsaturated fatty acids, exhibits pronounced antioxidant activity and strong inhibition of early-stage biofilm formation. Indian mustard oil, rich in erucic and α-linolenic acids and characterized by a balanced ω-6/ω-3 ratio, displays notable anti-arthritic and antioxidant properties, as well as significant antibiofilm activity. In contrast, avocado oil, dominated by monounsaturated fatty acids, shows superior activity against mature biofilms and marked cholinesterase-inhibitory effects. The integration of biochemical, microbiological, multivariate, and molecular docking analyses indicates that fatty acid composition may contribute to the multifunctional biological properties of these oils. Additionally, the capacity of these oils to stimulate the growth of selected probiotic strains while inhibiting biofilm formation by pathogenic bacteria underscores their potential as multifunctional ingredients that support microbial balance and health-promoting functions. These findings support the application of these seed oils as natural ingredients in food, nutraceutical, cosmetic, and health-related formulations. Specifically, their antioxidant, enzyme-inhibitory, antibiofilm, and probiotic growth-promoting properties suggest roles in the development of innovative functional products and sustainable value-added applications derived from agro-industrial by-products. However, certain limitations should be acknowledged. This study relied exclusively on *in vitro* assays, and the contribution of minor bioactive constituents, such as tocopherols, phytosterols, carotenoids, and phenolic compounds, was not specifically investigated. Consequently, the observed biological activities cannot be attributed solely to fatty acid composition, and additional compounds may also contribute to the reported effects. Future research should focus on detailed characterization of minor bioactive constituents, investigation of potential synergistic interactions among oil components, and validation of the observed biological activities in cellular and *in vivo* models. These approaches will enhance understanding of the mechanisms underlying the multifunctional properties of these oils and support their development as sustainable, high-value ingredients for applications in food, health, cosmetic, and industrial sectors.

## Data Availability

The original contributions presented in this study are included in this article/Supplementary material, further inquiries can be directed to the corresponding authors.

## Supplementary material

Supplementary Figure S1 Group of representative AChE docking visualizations generated for *Electrophorus electricus* acetylcholinesterase (PDB: 1EEA). Each letter identifies one ligand subset composed of paired 3D pose and 2D interaction panels: **(Aa,b)** acetylcholine, **(Ba,b)** acetylthiocholine, **(Ca,b)** alpha-linolenic acid, **(Da,b)** galantamine, and **(Ea,b)** nervonic acid. The 3D panels indicate ligand orientation within the AChE catalytic gorge, whereas the 2D diagrams identify recurrent residue contacts used to interpret the catalytic-gorge interactions reported in Table 3.

Supplementary Figure S2 BChE (PDB: 1P0I) docking visualizations. Group of representative BChE docking visualizations generated for human butyrylcholinesterase (PDB: 1P0I), used as the structural model for the BChE catalytic pocket. Each letter identifies one ligand subset composed of paired 3D pose and 2D interaction panels: **(Aa,b)** alpha-linolenic acid, **(Ba,b)** butyrylcholine, **(Ca,b)** butyrylthiocholine, and **(Da,b)** nervonic acid. The 3D panels show occupation of the BChE catalytic gorge/pocket, while the 2D interaction diagrams summarize the recurrent contacts with residues such as TRP82, GLU197, SER198, PHE329, TYR332, and HIS438.

Supplementary Figure S3 Tyrosinase (PDB: 3NQ1) docking visualizations. Group of representative tyrosinase docking visualizations generated for Bacillus megaterium tyrosinase (PDB: 3NQ1). Each letter identifies one ligand subset composed of paired 3D pose and 2D interaction panels: **(Aa,b)** dicopper-site alpha-linolenic acid, **(Ba,b)** dicopper-site kojic acid, **(Ca,b)** dicopper-site L-DOPA, **(Da,b)** dicopper-site L-tyrosine, **(Ea,b)** dicopper-site linoleic acid, **(Fa,b)** kojic-acid-like-site alpha-linolenic acid, and **(Ga,b)** kojic-acid-like-site kojic acid. These panels distinguish contacts near the dicopper environment from interactions in the adjacent pocket and support the interpretation that fatty-acid binding to tyrosinase is weaker and more site-variable than that observed for cholinesterases.
