# Supplementary figures and images for: Linking fatty acid composition to enzyme inhibition supported by molecular docking, alongside multifunctional bioactivities, of avocado, Indian mustard, and passion fruit seed oils
Source: Front Nutr. 2026 Jul 16;13:1874793. doi: 10.3389/fnut.2026.1874793 (PMC13422180; doi:10.3389/fnut.2026.1874793)

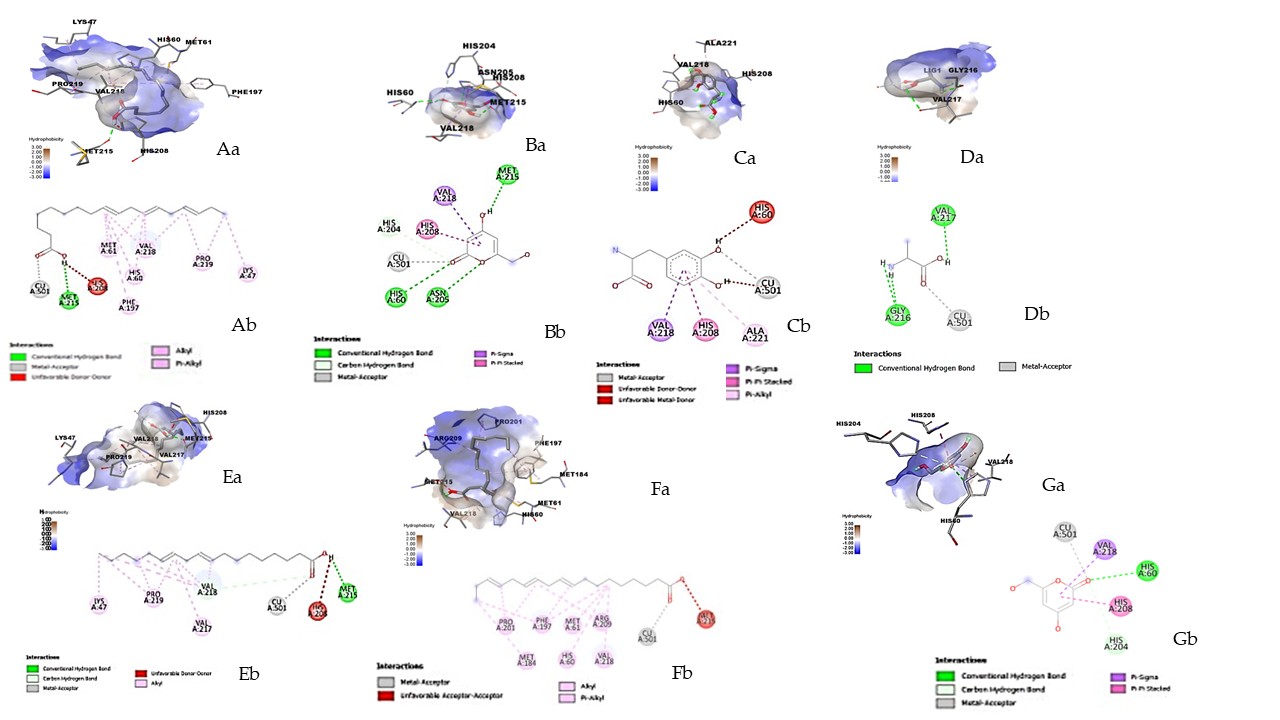

Supplement: Supplementary Figure S1 — Group of representative AChE docking visualizations generated for Electrophorus electricus acetylcholinesterase (PDB: 1EEA). Each letter identifies one ligand subset composed of paired 3D pose and 2D interaction panels: (Aa,b) acetylcholine, (Ba,b) acetylthiocholine, (Ca,b) alpha-linolenic acid, (Da,b) galantamine, and (Ea,b) nervonic acid. The 3D panels indicate ligand orientation within the AChE catalytic gorge, whereas the 2D diagrams identify recurrent residue contacts used to interpret the catalytic-gorge interactions reported in Table 3. [file Image_1.jpeg]

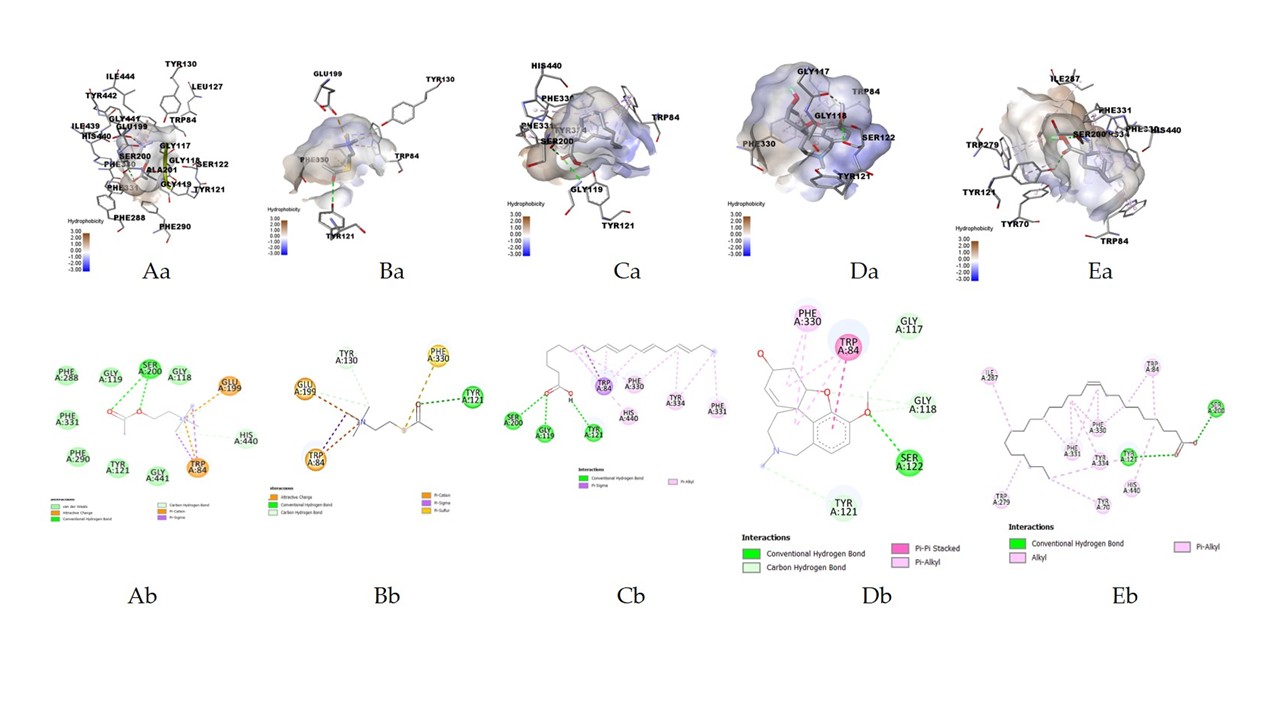

Supplement: Supplementary Figure S2 — BChE (PDB: 1P0I) docking visualizations. Group of representative BChE docking visualizations generated for human butyrylcholinesterase (PDB: 1P0I), used as the structural model for the BChE catalytic pocket. Each letter identifies one ligand subset composed of paired 3D pose and 2D interaction panels: (Aa,b) alpha-linolenic acid, (Ba,b) butyrylcholine, (Ca,b) butyrylthiocholine, and (Da,b) nervonic acid. The 3D panels show occupation of the BChE catalytic gorge/pocket, while the 2D interaction diagrams summarize the recurrent contacts with residues such as TRP82, GLU197, SER198, PHE329, TYR332, and HIS438. [file Image_2.jpeg]

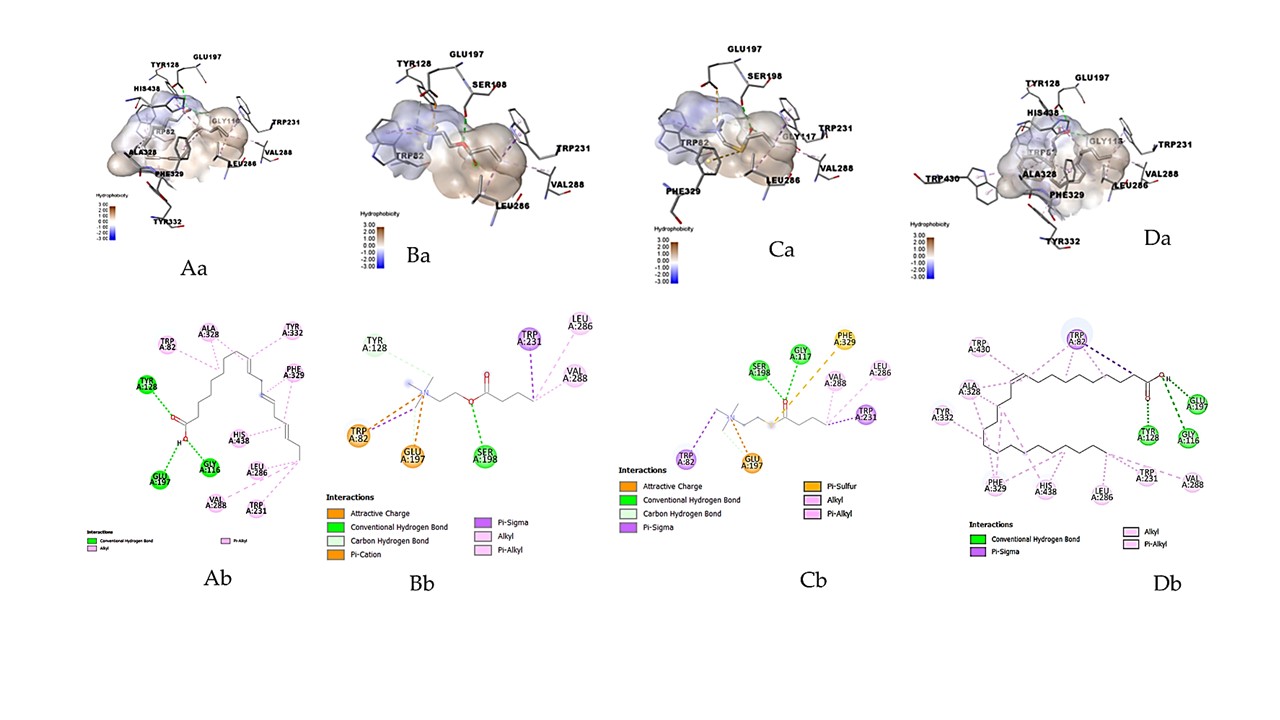

Supplement: Supplementary Figure S3 — Tyrosinase (PDB: 3NQ1) docking visualizations. Group of representative tyrosinase docking visualizations generated for Bacillus megaterium tyrosinase (PDB: 3NQ1). Each letter identifies one ligand subset composed of paired 3D pose and 2D interaction panels: (Aa,b) dicopper-site alpha-linolenic acid, (Ba,b) dicopper-site kojic acid, (Ca,b) dicopper-site L-DOPA, (Da,b) dicopper-site L-tyrosine, (Ea,b) dicopper-site linoleic acid, (Fa,b) kojic-acid-like-site alpha-linolenic acid, and (Ga,b) kojic-acid-like-site kojic acid. These panels distinguish contacts near the dicopper environment from interactions in the adjacent pocket and support the interpretation that fatty-acid binding to tyrosinase is weaker and more site-variable than that observed for cholinesterases. [file Image_3.jpeg]
